# From Early Intervention in Psychosis to Intensive Care: correlates of restrictive psychiatric practice in a national retrospective cohort study

**DOI:** 10.1192/bjp.2026.10637

**Published:** 2026-04-30

**Authors:** Ryan Williams, Edward Penington, Veenu Gupta, Michelle Rickett, Joel Agorinya, Apostolos Tsiachristas, Carolyn Chew-Graham, Jonathan Woodward, David Shiers, Alex Bottle, Benjamin McNeillis, Paul French, Belinda Lennox, Mike J Crawford

**Affiliations:** 1 Division of Psychiatry, Imperial College London, London, UK; 2 South London & The Maudsley NHS Foundation Trust, London, UK; 3 Department of Psychiatry, University of Oxford, Oxford, UK; 4 Department of Psychology, Durham University, Durham, UK; 5 School of Medicine, Faculty of Medicine and Health Sciences, Keele University, Newcastle, UK; 6 Accra Psychiatric Hospital, Accra, Ghana; 7 School of Medicine and Population Health, University of Sheffield, Sheffield, UK; 8 Department of Research and Innovation, Pennine Care NHS Foundation Trust, Lancashire, UK

**Keywords:** early intervention, psychosis, schizophrenia, clozapine, CBTp, psychiatric intensive care, restrictive practice

## Abstract

**Background:**

Restrictive interventions are used in the treatment of some people with severe mental disorders such as psychosis - including psychiatric intensive care unit (PICU) admission, seclusion and restraint. Early Intervention in Psychosis (EIP) service input may improve outcomes in psychosis, but it is unclear whether specific components of EIP care reduce the need for restrictive practice.

**Aims:**

To examine associations between EIP care components, demographic characteristics and restrictive interventions.

**Method:**

We conducted a retrospective cohort study of 14,874 EIP service users in England, using linked data from the National Clinical Audit of Psychosis and the Mental Health Services Data Set. We examined associations between EIP components and time to PICU admission (primary outcome) alongside seclusion/ physical restraint/ injected chemical restraint/ requests for police assistance (secondary outcomes) using multilevel Cox regression, adjusting for demographic factors and clustering by service.

**Results:**

Higher hazards of restrictive interventions were observed among men, younger people and several minority ethnic groups. Individuals eligible for clozapine who were not offered it (HR 1.51, 95% CI 1.20–1.91) or refused it (HR 1.46, 95% CI 1.02–2.10) had higher hazards of PICU admission than those not eligible, while those who were eligible for clozapine and received it did not. There was weaker evidence of similar effects on hazards of physical restraint and seclusion. Receipt of CBT for psychosis was associated with reduced hazards of PICU admission (HR 0.80, 95% CI 0.67–0.95) and physical restraint (HR 0.68, 95% CI 0.47–0.98). Substance use was associated with increased hazards of PICU admission and requests for police assistance, although substance-use interventions appeared to partially mitigate this.

**Conclusions:**

Marked demographic disparities exist in the use of restrictive practice. Specific EIP care components may be associated with reductions. Strengthening evidence-based EIP provision and addressing structural inequalities may support progress towards less coercive and more equitable care.

## Introduction

Psychotic disorders are common, with an estimated lifetime prevalence of 1-3% and an annual incidence of 20-30 per 100,000 person-years, and are associated with considerable morbidity.^1, 2^ A substantial minority of individuals with psychosis experience severe difficulties and will be treated at some stage with restrictive interventions - approximately 5-30% of those requiring inpatient care depending on setting and definition.^3, 4^ These are intended to manage immediate risk and include admission to psychiatric intensive care units (PICU) - tertiary mental health facilities for people in an acutely disturbed state, presenting with increased risk of harm to self or others.^5^ Other restrictive interventions include seclusion (‘supervised confinement and isolation… for the purpose of the containment of severe behavioural disturbance which is likely to cause harm’),^6^ physical or chemical restraint, and recourse to police assistance. Such interventions are controversial and understudied - a Cochrane review identified no controlled studies evaluating the value of seclusion or restraint in serious mental illness.^7^ They may also be traumatic and negatively affect therapeutic relationships, with many patients and carers describing them as frightening or disempowering.^8, 9^ Adverse mental health outcomes have been reported,^10, 11^ alongside concerns regarding resource use,^12^ workforce burden and the ethical legitimacy of coercive treatment.^13, 14^ In addition, there is well-documented demographic variation in their use, particularly affecting minority ethnic groups.^15–17^

Nevertheless, many clinicians view them as necessary (particularly those with greater exposure to aggressive behaviours).^18, 19^ They may be necessary to avert worse immediate outcomes such as serious self-harm or violence - healthcare staff are the most frequently assaulted professional group,^20^ with mental health nurses experiencing particularly high risk.^21^ Rather than an unwanted outcome, they may be better conceptualised as markers of illness severity, or pragmatic system-level responses to risk. Understanding how routine clinical practice may affect the use of restrictive interventions (and ideally reducing them) is therefore a priority for services and policymakers.^22^

Early Intervention in Psychosis (EIP) services aim to deliver a comprehensive package of evidence-based care to people experiencing psychosis.^23^ Their input has been associated with improved outcomes compared with standard care.^24^ However, it remains unclear which components of EIP provision are most beneficial. A recent study identified associations between specific components and hospital admission/ compulsory treatment,^25^ but their relations to restrictive interventions remain unknown. There is also increasing evidence that demographic characteristics may influence both the offer and uptake of components of EIP care.^26, 27^ Clarification is essential if EIP services are to mitigate, rather than mirror, inequities seen elsewhere. This retrospective cohort study of 14,874 individuals used linked national audit and routine data to examine associations between components of EIP care, demographic characteristics and subsequent restrictive psychiatric interventions, with the aim of identifying modifiable targets for more equitable and less coercive care.

## Method

This study followed the STROBE guidelines for observational studies.^28^ A full checklist is provided in the Appendix. This study was informed by service users and carers and their priorities for research.

### Study Design

We conducted a retrospective cohort study of 14,874 EIP service users using linked data from the National Clinical Audit of Psychosis (NCAP) and the Mental Health Services Data Set (MHSDS).

### Data sources and linkage

The NCAP is a quality improvement programme led by the Royal College of Psychiatrists that has collected patient-level data from all EIP services in England since 2017.^29^ We used data from the 2019/20 and 2020/21 rounds of the NCAP for this study, which were the first to obtain section 251 ethical approval to collect identifiable information (NHS number and date of birth) for the purpose of data linkage. These rounds included case-note audits conducted using a fixed census date (1^st^ April 2019 and 2020 respectively) - identifying eligible patients who had been on an EIP caseload for >6 months on the census date, and examining the components of care those patients had received during their time on the EIP caseload. During each round, a random sample of 100 eligible patients were selected from each service by the NCAP team using an Excel function (local services had no role in selecting patients). Where a service had a caseload with <100 eligible patients, the entire caseload was sampled. Information documented by treating clinicians was extracted from clinical records by trained local auditors following NCAP guidance.

In parallel, each audit round included a ‘contextual survey’ of team-level attributes (team caseload, number of care coordinators etc). Detailed accounts of the methods involved in the NCAP have been published elsewhere.^30^

For the purposes of this study, these data were linked to outcome data from MHSDS covering the period April 2016 to October 2022, providing two to three years of ‘follow-up’ after the NCAP census dates, depending on audit round.

NCAP records were deterministically linked to MHSDS by NHS Digital using personal identifiers (NHS number and date of birth) collected under section 251 approval. Personal identifiers were not accessed by the research team; instead, data were transferred directly by data controllers to the Office for National Statistics (ONS) Secure Research Service, where unique non-identifiable IDs were generated and used to link records across datasets and audit rounds. Personal identifiers were then removed, and all analyses were conducted on the resulting pseudonymised dataset.

88.9% of NCAP records were successfully linked to an MHSDS referral. Following de-duplication and exclusion of ambiguous matches, 14,874 unique individuals were included in the analytic cohort. Further details of linkage and data cleaning are provided in the Appendix.

### Participants

Eligibility for this study was determined upstream by inclusion in the NCAP (see Data sources and linkage). Inclusion criteria (for the NCAP, and by extension for this study) were:

- Clinical diagnosis of a ‘first episode’ of any ‘non-organic’ psychotic disorder, as determined by local clinical teams during NCAP eligibility assessment. The NCAP does not mandate use of a specific diagnostic framework (e.g. ICD or DSM), reflecting an intention to maximise inclusivity and real-world generalisability.
- Under EIP care for >6 months at NCAP audit census date.
- Aged 14-65 – reflecting national access standards.^31^

Individuals with psychosis due to an ‘organic cause’ were excluded from the NCAP – these were defined according to NCAP sampling guidance^30^ – possible examples given included neurodegenerative disorders (e.g. Huntington’s or Parkinson’s disease), HIV or syphilis, dementia, and intracranial tumours or cysts. Again, the use of a specific diagnostic framework was not required.

As eligibility criteria were applied as part of the NCAP audit process prior to sampling, information on individuals assessed but excluded at each eligibility stage was not available (auditors were not required to provide this).

### Exposure Variables

Our exposures were NICE-recommended components of EIP care:^32^ receipt of ‘cognitive behavioural therapy for psychosis’ (CBTp), family intervention, vocational support, and carer-focused interventions; offer and initiation of clozapine where appropriate (patients were eligible if they had ‘treatment-resistant’ symptoms i.e. inadequate response to two previous antipsychotics), receipt of NICE-approved EIP physical health interventions (these were grouped into interventions for smoking/ alcohol/ psychoactive substance cessation, or weight reduction). Service-level exposures were average care coordinator caseload size per service, and waiting time (proportion of those at each service meeting waiting time standards).

All exposure variables relating to EIP care were derived from the NCAP case-note review and contextual surveys and were not determined by the study investigators. These variables reflect information documented by treating clinicians and extracted by trained local auditors using standardised NCAP guidance. Exposures were pre-specified by the structure of the NCAP, reflecting clinical and policy grounds; no data-driven variable selection was involved.

### Outcome Variables

All outcome variables were derived from the MHSDS. Our primary outcome was time to PICU admission. Secondary outcomes were time to incidents of seclusion, physical restraint, injected chemical restraint and requests for police assistance, as defined as distinct restrictive intervention categories within MHSDS.^33^ A further planned secondary outcome (time to incident of oral chemical restraint) was not included because the number of recorded incidents was small, likely reflecting that this was not well-recorded in electronic records. Further details of variable provenance and derivation are provided in the Appendix.

### Statistical Analyses

Analyses were performed using ‘R’.^34^ Initially we generated descriptive statistics for all exposure variables, outcome measures and covariates, and explored unadjusted associations between each exposure, covariate and outcome using single-predictor regression models.

Next, we conducted multivariable analyses using Cox regression. Models examined time to first recorded event during follow-up for each outcome. To account for clustering of individuals within services, we fitted mixed-effects Cox models including a random intercept for EIP service, with all exposures and covariates entered as fixed effects.

The time of exposure was taken as the relevant NCAP audit census date for each individual (1st April 2019 or 1st April 2020). Participants were censored at death or at the end of available follow-up in the MHSDS. Individuals who died during follow-up were not excluded from analyses - deaths were treated as censoring events rather than as competing risks as the number observed was small (n = 201). There were no additional censoring variables i.e. all participants were followed up to the outcome of interest or the end of follow up. Multilevel models were used to account for clustering effects within EIP services and were adjusted for covariates including age, sex, ethnicity and employment status.

Results were also adjusted for whether individuals had already required PICU admission prior to EIP involvement (within the data extract window, i.e. post-April 2016), and their number of non-PICU admissions in the follow-up period. For the secondary outcomes ‘injected chemical/ physical restraint’ and ‘seclusion’, analyses were restricted to only those with a hospital admission or PICU admission respectively as these outcomes could only occur in these settings.

Model development followed an information-criterion approach. Exposure variables of interest were prespecified and retained across models. Demographic and clinical covariates were evaluated in candidate models and retained in final models where they contributed to model fit, assessed using Akaike Information Criterion (AIC). We formally assessed model assumptions, including proportional hazards, using appropriate statistical tests (see Appendix for further details of statistical methods).

Given that clozapine has a clearly defined indication for treatment-resistant illness, we conducted a supplementary sensitivity analysis of the primary outcome restricted to individuals recorded as eligible for clozapine. Model specification, covariate adjustment and handling of clustering were otherwise identical to the primary analyses. This approach was undertaken to facilitate interpretation of the effect of clozapine solely within the clinically indicated population and to examine whether the pattern observed in the full cohort was consistent when restricted to those meeting eligibility criteria.

### Missing Data

Data for chosen outcomes are a combination of ‘mandatory’ and ‘required’ submissions for NHS England and undergo a range of data validation checks as part of submission. Absence of a record was treated as absence of the event. There is a strong possibility that in some cases, missing data was due to under-recording rather than a true absence. For the primary outcome (PICU admission), we feel this possibility is small due to it being a mandatory submission. For secondary outcomes, this was likely the case to some degree as counts were lower than expected (e.g. only 125/712 or 17.6% of those admitted to PICU in our sample had experienced injected chemical restraint, compared to a reported rate of 32% in another recent study).^35^ While such differential under-recording of secondary outcomes across groups could bias estimated associations, it was not possible to formally test this.

For a small number of cases (<0.5% of those included in NCAP), linkage to MHSDS was not possible due to no eligible ‘referral’ recorded in MHSDS (see Appendix for summary of linkage process).

## Results

Data were obtained for 14,874 participants. Mean age was 32.2 years (SD 11.4), and 9225 were male (62.0%). Demographic characteristics and a summary of exposure and outcome variables are given in Table 1, with more detailed breakdowns (specific ethnicity categories) available in the Appendix.

### Primary Outcome – PICU admission

Associations between exposure variables, covariates and adjusted hazard rates of PICU admission (i.e. the risk of an admission occurring at any given time point during the follow-up period) are reported in Table 2. Hazard ratios indicate the relative rate at which participants with a given exposure experienced the outcome over follow-up, compared with the reference category for each variable. Values >1 indicate a higher rate of the outcome, while values <1 indicate a lower rate, relative to the reference group. Overall (unstratified) absolute time-to-event summaries for primary and secondary outcomes are also shown in Table A6 in the Appendix.

We found strong evidence that demographic characteristics were associated with altered rates of admission to PICU. Rates were higher for men than women (HR 1.86, 95% CI 1.56 to 2.21, p<0.001), and lower for all age groups older than 25-34. Compared to those of White ethnicity, hazard rates of PICU admission were higher for people identified as Black/Black British (HR 1.41, 95% CI 1.16 to 1.71, p=0.001), Mixed ethnicity (HR 1.61, 95% CI 1.21 to 2.14, p=0.001), Other ethnicity (HR 1.83, 95% CI 1.28 to 2.60, p=0.001) and Unknown/ Undocumented ethnicity (HR 1.76, 95% CI 1.07 to 2.91, p=0.027).

Individuals who had already had a PICU admission prior to the start of their episode of EIP care had substantially higher hazard rates of subsequent admission compared with those who had not (HR 3.98, 95% CI 3.40 to 4.67, p<0.001), and rates of PICU admission were much higher in those with increasing numbers of non-PICU admissions in the follow-up period – those with 1-2 admissions (HR 3.64, 95% CI 3.08 to 4.30, p<0.001) and those with 3+ (HR 6.49, 95% CI 5.24 to 8.05, p<0.001).

We also found strong evidence that receipt of CBTp and the use of clozapine were associated with altered rates of admission to PICU. Those who received CBTp had reduced hazard rates compared to those who were not offered it (HR 0.80, 95% CI 0.67 to 0.95, p=0.012), while those who were offered CBTp and refused it had increased rates (HR 1.22, 95% CI 1.01 to 1.47, p=0.040). For clozapine, we found substantially increased hazard rates (compared with those who were not eligible to receive it) for those who were eligible to receive it and refused it (HR 1.46, 95% CI 1.02 to 2.10, p=0.040), or who were not offered it (HR 1.51, 95% CI 1.20 to 1.91, p<0.001). However, those who were eligible for clozapine and received it showed no significant difference to ineligible individuals (HR 1.08, 95% CI 0.82 to 1.43, p=0.590).

Hazard rates were higher for those requiring substance use interventions, regardless of whether interventions were refused (HR 1.71, 95% CI 1.38 to 2.13, p<0.001) or received (HR 1.48, 95% CI 1.23 to 1.78, p<0.001) – although rates appeared lower among those who received interventions than among those who refused them, these differences were not formally tested. No other components of care were associated with significant differences in rates of PICU admission.

### Secondary Outcomes

**Figure 1:**
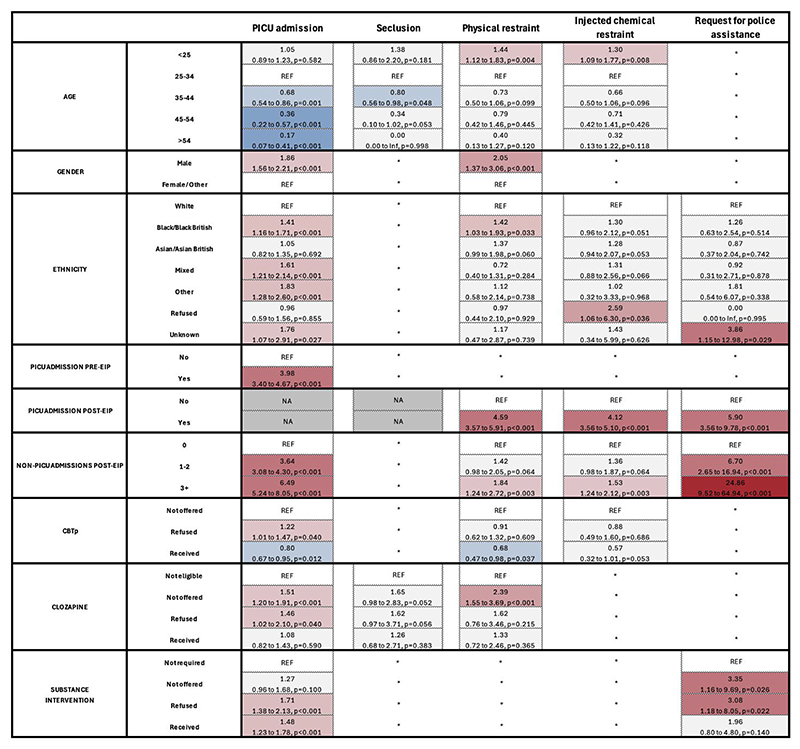
Heat map of significant associations from final adjusted models for all outcomes. This figure presents a heat map summarising adjusted hazard ratios (HRs) from the final multivariable Cox models for all primary and secondary outcomes. Each cell displays the HR with 95% confidence interval and p-value, comparing each exposure category with its designated reference group while adjusting for demographic and clinical covariates and clustering within services. Included demographic and clinical covariates are those retained in the final models for each outcome following evaluation in candidate models, due to improving model fit - as described in the Methods and Table 2. Colour shading reflects the magnitude and direction of effect sizes for statistically significant differences: red tones indicate HRs >1 (increased likelihood of the outcome compared to the reference category), and blue tones indicate HRs <1 (reduced likelihood). Darker intensity corresponds to larger effect sizes. Some cells are blank (*) because these variables were not included in the final model for that outcome. Some are restricted (NA) because those variables were conceptually incompatible with the outcome. For example, post-EIP PICU admission cannot be modelled as a predictor of itself, and seclusion is only recorded among people already admitted to PICU.

Associations between exposure variables, covariates and adjusted hazard rates for secondary outcomes (incidents of seclusion, physical restraint, injected chemical restraint, and requests for police assistance) are reported in Tables A2-A5 in the Appendix. Results for all outcomes are summarised in a heat map in Figure 1 (showing the outputs for variables included in the final adjusted models for each outcome only).

#### Seclusion

For incidents of seclusion (among those who were admitted to PICU specifically), hazard rate changed with age – compared to those aged 25-34, rates were higher for those aged <25 and lower for older age groups.

There was also weak evidence that the use of clozapine was associated with altered rates. Specifically, there was weak evidence that again rates were increased for those who were eligible to receive it and refused it (HR 1.62, 95% CI 0.97 to 3.71, p=0.060), or who were not offered it (HR 1.65, 95% CI 0.98 to 2.83, p=0.052), while those who were eligible for clozapine and received it showed no significant difference to ineligible individuals (HR 1.26, 95% CI 0.68 to 2.71, p=0.383). No other components of care were associated with significant differences in rates of seclusion among those admitted to PICU.

For all of the following secondary outcomes - incidents of physical restraint, injected chemical restraint, and requests for police assistance – there was strong evidence that rates were increased for those with increasing numbers of admissions to hospital, and those who required PICU admission post-EIP involvement. However, even adjusting for these findings there was evidence of altered rates with specific demographics and components of care.

#### Physical restraint

For incidents of physical restraint (among those who were admitted to hospital), rates were higher for men than women (HR 2.05, 95% CI 1.37 to 3.06, p<0.001), and for those aged <25 compared to those aged 25-34 (HR 1.44, 95% CI 1.12 to 1.83, p=0.004). People identified as Black/ Black British had higher hazard rates of physical restraint than those of White ethnicity (HR 1.42, 95% CI 1.03 to 1.93, p=0.033).

We also found evidence that receipt of CBTp and clozapine were associated with altered rates of physical restraint. Those who received CBTp had reduced hazard rates compared to those who were not offered it (HR 0.68, 95% CI 0.47 to 0.98, p=0.037), while there was no significant difference for those who were offered it and refused it. For clozapine, the rate was significantly increased for those who were eligible to receive it and were not offered it (HR 2.39, 95% CI 1.55 to 3.69, p<0.001) – but there were no significant differences between those who refused it, received it, or were ineligible. No other components of care were associated with significant differences in rates of physical restraint.

#### Injected chemical restraint

For injected chemical restraint (among those who were admitted to hospital), again rates were higher for those aged <25 compared to those aged 25-34 (HR 1.30, 95% CI 1.09 to 1.77, p=0.008). Higher hazard rates were also observed for people who refused to disclose their ethnicity, compared to those of White ethnicity (HR 2.59, 95% CI 1.06 to 6.30, p=0.036), with additional weak evidence of higher rates in those identified as Black/ Black British (HR 1.30, 95% CI 0.96 to 2.12, p=0.051) and possibly also Asian/ Asian British (HR 1.28, 95% CI 0.94 to 2.07, p=0.053).

We also found weak evidence that receipt of CBTp was associated with altered rates of chemical restraint. Specifically, there was weak evidence that those who received CBTp had reduced hazard rates compared to those who were not offered it (HR 0.57, 95% CI 0.32 to 1.01, p=0.053), while there was no significant difference for those who refused it. No other components of care were associated with significant differences in rates of injected chemical restraint.

#### Request for police assistance

For requests for police assistance, people whose ethnicity was recorded as Unknown/ Undocumented had higher hazard rates than people of White ethnicity (HR 3.86, 95% CI 1.15 to 12.98, p=0.029).

There was also evidence that interventions for substance use were associated with altered rates. Specifically, we found substantially increased hazard rates for those who were eligible for interventions for substance use and refused them (HR 3.08, 95% CI 1.18 to 8.05, p=0.022), or who were not offered them (HR 3.35, 95% CI 1.16 to 9.69, p=0.026). However, those who required interventions for substance use and received them showed a smaller (non-significant) difference compared to ineligible individuals (HR 1.96, 95% CI 0.80 to 4.80, p=0.140). No other components of care were associated with significant differences in rates of requests for police assistance.

### Sensitivity Analysis

The results of the sensitivity analysis restricted to individuals recorded as eligible for clozapine are reported in Table A7 in the Appendix. Overall, patterns of association were broadly consistent with those observed in the full cohort. Within this subgroup, individuals who were eligible for clozapine and received it had lower hazards of PICU admission than those who were eligible but not offered it (HR 0.71, 95% CI 0.45–0.94, p=0.039). Refusal of clozapine was not significantly associated with altered hazard. Other exposures showed similar directions of effect to the main analysis. However, in contrast to the full cohort, receipt of CBTp was not associated with reduced hazard of PICU admission among those eligible for clozapine.

## Discussion

This study is the first to examine restrictive psychiatric interventions using population-level routine outcome data in the UK. By linking the NCAP and MHSDS we were able to examine a large, nationally representative cohort and quantify associations between components of EIP care, demographic characteristics, and subsequent use of restrictive interventions. If these associations reflect causal mechanisms, they suggest that known variation in the delivery of evidence-based EIP components may contribute to downstream disparities in restrictive practice, with implications for service commissioning, policy and clinical practice.

We identified consistent associations between specific care components and the likelihood of PICU admission, seclusion, chemical/ physical restraint and requests for police assistance. As outlined in the introduction, these restrictive interventions can represent necessary and therapeutic responses to acute risk. Associations with lower rates should therefore be interpreted as reflecting differences in severity or service response rather than as implying that such interventions are inherently undesirable; nonetheless, reducing their use where clinically appropriate and safely achievable remains an important service and policy objective.

Clozapine treatment for those eligible was associated with more favourable outcomes than non-initiation or refusal for PICU admission and physical restraint (with weaker evidence for rates of seclusion). Receipt of CBTp was associated with reduced rates of PICU admission and physical restraint (with weaker evidence for reduced rates of injected chemical restraint). Interventions for substance use appeared to partially mitigate the elevated risks of PICU admission and requests for police assistance associated with comorbid substance misuse. Demographic disparities persisted across most outcomes, with higher hazard rates among men, younger individuals, and people from minority ethnic groups. Prior service use (earlier PICU admission and repeated hospitalisation) was among the strongest predictors of subsequent restrictive intervention. A notable negative finding was that family interventions were not associated with reduced risk of any restrictive psychiatric intervention despite a relatively strong evidence base for improved outcomes in early psychosis more generally.

Our findings suggest there may be clinically meaningful benefits with CBTp. The association with reduced PICU admission was not observed among those who were offered therapy and refused it. This suggests a reduced likelihood of ‘confounding by indication’ (e.g. selective offering to those at low risk of experiencing restrictive interventions) – unless clinicians routinely recorded refusal for patients who were too unwell to consent. Residual confounding related to engagement or perceived capacity to benefit cannot be excluded - i.e. those more likely to engage with care may have been more likely to receive CBTp rather than refuse or not be offered (as the decision not to offer may reflect clinicians’ expectations regarding engagement) - and also less likely to experience restrictive interventions. However, CBTp may itself enhance engagement with care, through improved coping skills and insight, or strengthened therapeutic alliance with clinicians. Adjustment for prior hospitalisation and PICU use also addressed confounding by illness severity. These findings are consistent with previous work linking CBTp to reduced rates of detention, if not reduced relapse or hospital admission.^25^ It is possible that while CBTp may not avert relapse, it may influence the manner in which relapse manifests or is managed - avoiding escalation to circumstances requiring restrictive intervention. These benefits may not extend to reduced behavioural disturbance requiring restrictive intervention, or variability in the delivery of family interventions may have limited their impact on these outcomes in routine practice

Findings relating to clozapine align with a strong evidence base,^25, 36, 37^ including trials suggesting reductions in aggressive behaviour among people with psychosis.^38, 39^ Importantly, all interpretations relating to clozapine in this study apply only to individuals recorded as eligible for clozapine on the basis of treatment resistance. Eligible individuals who were not offered or who refused clozapine had substantially higher risks of PICU admission than those who received it, even after adjustment for previous PICU admission and overall admission rate. Supplementary analyses restricted to the eligible subgroup yielded consistent patterns of association, supporting the robustness of these findings within the clinically indicated population (see Appendix).

As with CBTp, confounding by treatment adherence or engagement cannot be fully excluded. Those more likely to engage with care may have been more likely to receive clozapine rather than refuse or not be offered (if the decision not to offer was related to anticipated non-adherence). However, it is equally possible that failure to offer clozapine reflects other service-level barriers – in which case the contrast with those who received it would indicate a clinically meaningful protective effect. Such barriers are widely recognised - treatment resistance is likely under-identified in early psychosis, and many individuals eligible for clozapine do not receive it.^40, 41^ It is notable that clozapine was the only component associated (albeit weakly) with reduced rates of seclusion once admitted to PICU, suggesting potential benefits even among those requiring high-intensity care. Our findings reinforce the need for strategies to improve timely identification of treatment resistance, reduce barriers to clozapine initiation, and support adherence during early psychosis.

Substance use emerged as a correlate of restrictive intervention (PICU admission and police assistance), consistent with previous evidence that substance misuse may be an issue for the majority of those requiring PICU.^42^ However, receipt of interventions to reduce substance use was associated with lower hazard rates than refusal, indicating some possible mitigating effects. This underscores the importance of integrated dual-diagnosis care not only within EIP but across service settings.

Persistent racial and demographic disparities in our data echo longstanding concerns regarding disproportionate use of coercive practices.^15, 16, 43^ Higher hazard rates were observed among minority ethnic groups (PICU admission, physical and chemical restraint, and requests for police assistance), younger age groups (PICU admission, seclusion, physical and chemical restraint) and men (PICU admission and physical restraint). The fact that disparities emerged even within an EIP cohort highlights the limits of this model alone in counteracting broader structural drivers, and reinforces the need for sustained efforts to address structural inequalities including culturally responsive care, staff training and co-production with minoritised communities to redesign acute pathways.

Finally, we found that patterns of prior service use were consistently among the strongest predictors of subsequent restrictive intervention. Individuals with previous PICU admission or frequent hospitalisation had markedly higher risks of further restrictive intervention. While this likely reflects underlying illness severity and complexity, it may also capture the cumulative detrimental effects of coercive experiences, such as trauma, lack of therapeutic alliance and reduced engagement, that may increase the likelihood of escalation in a self-reinforcing cycle. This interpretation highlights the potential long-term implications of early negative experiences of mental health service use.

### Strengths and limitations

We used a large, nationally representative cohort and high-quality datasets with robust validation procedures. The linkage of NCAP and routine outcome data enabled comprehensive modelling of real-world patterns of restrictive practice using multilevel time-to-event analyses.

Limitations include the observational design and potential residual confounding. We were not able to include factors such as engagement with care and adherence to medication, which are known to increase the likelihood of admission to hospital among people with psychosis.^44^ Physical aggression is associated with admission to PICU units among people with psychosis,^45^ and levels of physical aggression may also have influenced whether people were offered the different components of EIP care examined in this study. We cannot be sure individuals not offered interventions such as CBTp could have meaningfully engaged had it been available, nor whether all individuals recorded as having refused had capacity to make this decision (refusal may in some cases reflect acute illness). Consequently, despite adjustment for prior admission and PICU use, delivery of some components may still have varied systematically by unmeasured illness severity.

For exposures contingent on clinical need (e.g. clozapine; substance-use interventions), comparisons to a ‘not eligible/not required’ reference group partly capture the adverse prognosis associated with the underlying indication, in addition to any association with intervention receipt. In particular, eligibility for clozapine reflects treatment-resistant illness and therefore denotes a group at intrinsically elevated risk. Although this framing provides clinically informative prognostic context, it complicates interpretation of between-group contrasts and may amplify apparent differences relative to those not meeting eligibility criteria. To clarify interpretation for clozapine specifically, we conducted supplementary analyses restricted to individuals recorded as eligible.

Although we accounted for clustering by clinical service using multilevel models, this approach treats between-service variation as a random effect and does not permit direct estimation of service-specific associations. Variation between services may reflect structured differences in resources, staffing and local practice rather than random fluctuation alone. Alternative analytic strategies - such as service-stratified analyses using meta-analytic methods - may provide additional insight into the nature and sources of heterogeneity between services. Exploration of these approaches was beyond the scope of the present analysis but represents an important direction for future research.

Time-to-event measures were anchored to the NCAP audit census date rather than the precise timing of exposures (which were not available from the NCAP or MHSDS data). As a result, while some individuals may have completed components such as CBTp before the audit period, others may have still been mid-treatment. In a cohort of this size, such variability is likely to distribute randomly and thus attenuate rather than inflate observed associations, and is unlikely to differ systematically between exposure categories.

Year of study entry was not modelled as a fixed effect, despite representing a non-random source of variation associated with the COVID-19 pandemic and related changes in service provision. As a result, our estimates should be interpreted as averaged across audit years and may obscure heterogeneity in associations between pre-pandemic and pandemic periods. Year-stratified analyses or meta-analytic approaches could provide complementary insight into the effects of the pandemic but were beyond the scope of the present study.

As stated under ‘Missing Values’, the reported numbers for secondary outcomes were smaller than expected, and we are unable to identify whether these are true findings or whether data may be missing (or for that matter exclude the possibility that there may be data ‘missing not at random’, with the potential to bias results for these outcomes).

Statistical power limited our ability to explore heterogeneity within groups - for example, differential experiences among specific ethnic categories. Finally, generalisability may be constrained to settings with similar commissioning and policy frameworks to those in England, particularly given the national mandate and resourcing of EIP services.

### Conclusions

Taken together, these findings highlight the potential for specific components of EIP care to reduce the likelihood of restrictive intervention and point towards practical avenues for service improvement. As this was an observational study, the associations reported should not be interpreted as clear evidence of causal effects and may be influenced by residual confounding related to illness severity, engagement with care, or other unmeasured factors. However, ensuring timely access to CBTp and optimising pathways for the initiation of clozapine among individuals meeting criteria for treatment resistance may offer realistic, modifiable means of reducing escalation to restrictive practice. The potential mitigating effects of substance use interventions emphasise the need for fully integrated dual-diagnosis provision across mental health service settings. Persistent demographic disparities, and the strong predictive value of earlier restrictive experiences, underscore the importance of wider structural reforms, including culturally responsive models of care and collaborative approaches with communities most affected by coercive pathways.

Future research should examine causal mechanisms and evaluate targeted interventions to reduce restrictive practice. The former could involve (for example) prospective longitudinal studies examining whether improvements in specific CBTp domains (coping strategies, safety planning, mentalisation) mediate the relationship with restrictive practice. Qualitative research could also be useful to examine healthcare practitioners’ decision-making around restrictive practices, and the experiences and perspectives of patients who have received restrictive interventions - particularly those from high-risk groups. The latter could involve implementation trials of optimisation pathways in EIP – systematic screening for clozapine eligibility and streamlined titration clinics, or embedding substance use clinics within teams – perhaps with a particular focus on ‘at-risk’ groups including those with prior experience of PICU or repeated admissions. The development and evaluation of such interventions should meaningfully involve people with lived experience of psychosis, as well as families and carers, particularly those with prior experience of restrictive interventions. Together, these approaches offer a pathway towards less coercive and more equitable care for people experiencing psychosis.

## Supplementary Material

Supplementary Material

## Figures and Tables

**Table 1:** Cohort characteristics This table presents summarised demographics, exposures and outcomes for the cohort (N=14874). Note that for age, inter-quartile range (IQR) is reported rather than full range reported to avoid potentially identifiable data as per ONS SRS disclosure control requirements. For gender, the small number of individuals recorded in the ‘Other’ category were combined with ‘Female’, also in accordance with ONS SRS requirements. This approach was adopted following discussion with the study’s PPI group, who advised against exclusion of these individuals where possible.

DEMOGRAPHIC VARIABLES	
Age (%)	
<25	4944 (33.2)
25-34	2411 (16.2)
35-44	5203 (35.0)
45-54	1486 (10.0)
>54	830 (5.6)
Mean (SD)	32.2 (11.4)
Median (IQR)	28.8 (22.8 – 37.8)
Gender (%)	
Male	9225 (62.0)
Female (or other)	5649 (38.0)
Ethnicity (%)	
White	9627 (64.7)
Black/ Black British	1796 (12.1)
Asian/ Asian British	1768 (11.9)
Mixed	590 (4.0)
Other	507 (3.4)
Refused	348 (2.3)
Unknown/ Undocumented	238 (1.6)
In employment or education (%)	
No	8953 (60.2)
Yes	5921 (39.8)
EXPOSURE VARIABLES	
SERVICE LEVEL	
CARE COORDINATOR CASELOAD	
(number of patients per care coordinator at treating EIP service)	
Mean (SD)	18.5 (5.7)
Min-Max	7.0 − 54.5
Median (IQR)	17.4 (15.1 − 20.4)
PROPORTION WAITING TIME STANDARD MET	
(percentage of patients commencing treatment within 2 weeks at treating EIP service)	
Mean (SD)	74.7 (14.4)
Min-Max	30 - 100
Median (IQR)	75.0 (64.0 − 86.0)
PATIENT LEVEL	
RECEIVED CLOZAPINE (%)	
Not eligible	12964 (87.2)
Not offered	987 (6.7)
Refused	291 (1.9)
Yes	630 (4.2)
RECEIVED CBTp (%)	
No	3646 (24.5)
Refused	3975 (26.7)
Yes	7253 (48.8)
RECEIVED FAMILY INTERVENTION (%)	
No	6033 (40.6)
Refused	5508 (37.0)
Yes	3333 (22.4)
RECEIVED CARER INTERVENTION (%)	
Not eligible	4021 (27.0)
No	4724 (31.8)
Yes	6129 (41.2)
RECEIVED EMPLOYMENT INTERVENTION (%)	
No	5838 (39.2)
Refused	4160 (28.0)
Yes	4876 (32.8)
RECEIVED SMOKING INTERVENTION (%)	
Not required	7278 (48.9)
No	1512 (10.2)
Refused	2386 (16.0)
Yes	3698 (24.9)
RECEIVED WEIGHT INTERVENTION (%)	
Not required	8730 (58.7)
No	487 (3.3)
Refused	465 (3.1)
Yes	5192 (34.9)
RECEIVED ALCOHOL INTERVENTION (%)	
Not required	11682 (78.5)
No	1377 (9.3)
Refused	975 (6.6)
Yes	840 (5.6)
RECEIVED SUBSTANCE INTERVENTION (%)	
Not required	10005 (67.3)
No	1243 (8.4)
Refused	1327 (8.9)
Yes	2299 (15.4)
OUTCOME VARIABLES	
PICU ADMISSION (post-EIP)	
Yes (%)	712 (4.8)
No (%)	14162 (95.2)
SECLUSION (for those who were admitted to PICU only)	
Yes (%)	109 (15.3)
No (%)	603 (84.7)
PHYSICAL RESTRAINT (for those who were admitted to PICU or general acute wards)	
Yes (%)	336 (10.0)
No (%)	3013 (90.0)
INJECTED CHEMICAL RESTRAINT (for those who were admitted to PICU or general acute wards)	
Yes (%)	125 (3.7)
No (%)	3224 (96.3)
REQUESTS FOR POLICE ASSISTANCE (of those who were admitted to PICU or general acute wards)	
Yes (%)	68 (2.0)
No (%)	3281 (98.0)

**Table 2:** Associations between exposures and PICU admission (primary outcome) This table presents the unadjusted and adjusted hazard ratios (HRs) with 95% confidence intervals (CIs) for the primary outcome (PICU admission). The ‘Full Model’ includes all exposure variables and covariates, while the ‘Final Model’ is based on a refined selection of variables informed by statistical and theoretical considerations. Hazard ratios represent the relative likelihood of PICU admission occurring at any given time for individuals in one category of a variable compared with the reference category, holding all other variables constant. HR > 1 indicates an increased likelihood of PICU admission, while HR < 1 indicates a decreased likelihood. Results are adjusted for clustering within services. Results in **bold** indicate p-values ≤0.05.

Variables	Unadjusted HR (95% CI)	Adjusted HR - Full Model (95% CI)	Adjusted HR - Final Model (95% CI)
Age			
<25	1.12 (0.97 to 1.31, p=0.126)	1.05 (0.87 to 1.25, p=0.621)	1.05 (0.89 to 1.23, p=0.582)
25-34	Ref	Ref	Ref
35-44	**0.46 (0.37 to 0.57, p<0.001)**	**0.71 (0.55 to 0.91, p=0.007)**	**0.68 (0.54 to 0.86, p=0.001)**
45-54	**0.19 (0.12 to 0.29, p<0.001)**	**0.41 (0.25 to 0.66, p<0.001)**	**0.36 (0.22 to 0.57, p<0.001)**
>54	**0.11 (0.06 to 0.21, p<0.001)**	**0.18 (0.07 to 0.48, p=0.001)**	**0.17 (0.07 to 0.41, p<0.001)**
Sex			
Female (or other)	Ref	Ref	Ref
Male	**2.66 (2.24 to 3.16, p<0.001)**	**1.56 (1.27 to 1.92, p<0.001)**	**1.86 (1.56 to 2.21, p<0.001)**
Ethnicity			
White	Ref	Ref	Ref
Black/ Black British	**2.27 (1.90 to 2.71, p<0.001)**	**1.55 (1.24 to 1.94, p<0.001)**	**1.41 (1.16 to 1.71, p=0.001)**
Asian/ Asian British	1.13 (0.90 to 1.41, p=0.297)	1.09 (0.84 to 1.43, p=0.519)	1.05 (0.82 to 1.35, p=0.692)
Mixed	**2.32 (1.77 to 3.05, p<0.001)**	**1.80 (1.33 to 2.44, p<0.001)**	**1.61 (1.21 to 2.14, p=0.001)**
Other	**2.01 (1.44 to 2.79, p<0.001)**	**2.05 (1.41 to 2.99, p<0.001)**	**1.83 (1.28 to 2.60, p=0.001)**
Refused	**1.44 (0.94 to 2.22, p=0.093)**	1.13 (0.68 to 1.89, p=0.632)	0.96 (0.59 to 1.56, p=0.855)
Unknown/ Undocumented	**1.94 (1.24 to 2.04, p=0.004)**	**1.92 (1.11 to 3.30, p=0.019)**	**1.76 (1.07 to 2.91, p=0.027)**
Patient in employment or education			
No	Ref	Ref	
Yes	**0.85 (0.74 to 0.98, p=0.024)**	0.98 (0.83 to 1.17, p=0.830)	
PICU admission prior to EIP involvement			
No	Ref	Ref	Ref
Yes	**7.57 (6.59 to 8.69, p<0.001)**	**3.77 (3.17 to 4.49, p<0.001)**	**3.98 (3.40 to 4.67, p<0.001)**
Number of non-PICU admissions following EIP involvement			
0	Ref	Ref	Ref
1-2	**4.84 (4.11 to 5.70, p<0.001)**	**4.03 (3.36 to 4.84, p<0.001)**	**3.64 (3.08 to 4.30, p<0.001)**
3+	**10.76 (8.76 to 13.22, p<0.001)**	**7.43 (5.88 to 9.37, p<0.001)**	**6.49 (5.24 to 8.05, p<0.001)**
Average care coordinator caseload at treating EIP service*			
	**1.01 (1.00 to 1.01, p=0.046)**	**1.01 (1.01 to 1.02, p=0.059)**	
Likelihood that treatment began in <2 weeks (based on proportion meeting waiting time standard at treating service)*			
	1.00 (0.99 to 1.00, p=0.072)	1.00 (0.99 to 1.00, p=0.787)	
Received Cognitive Behavioural Therapy for Psychosis			
No	Ref	Ref	Ref
Refused	1.16 (0.96 to 1.39, p=0.132)	0.99 (0.78 to 1.26, p=0.952)	**1.22 (1.01 to 1.47, p=0.040)**
Yes	**0.74 (0.62 to 0.88, p=0.001)**	0.95 (0.76 to 1.19, p=0.674)	**0.80 (0.67 to 0.95, p=0.012)**
Received Family Intervention			
No	Ref	Ref	
Refused	1.08 (0.92 to 1.27, p=0.331)	0.95 (0.77 to 1.16, p=0.599)	
Yes	1.04 (0.87 to 1.24, p=0.676)	0.90 (0.72 to 1.13, p=0.382)	
Received carer-focussed intervention			
Not eligible	Ref	Ref	
No	**1.39 (1.15 to 1.67, p=0.001)**	1.08 (0.87 to 1.35, p=0.479)	
Yes	**1.34 (1.12 to 1.60, p =0.001)**	0.99 (0.80 to 1.24, p=0.964)	
Received vocational support			
No	Ref	Ref	
Refused	**1.24 (1.04 to 1.48, p=0.014)**	0.95 (0.77 to 1.19, p=0.669)	
Yes	**1.33 (1.13 to 1.56, p=0.001)**	1.11 (0.91 to 1.34, p=0.312)	
Received clozapine			
Not eligible	Ref	Ref	Ref
Not offered	**1.90 (1.51 to 2.39, p<0.001)**	1.30 (0.99-1.70, p=0.059)	**1.51 (1.20 to 1.91, p<0.001)**
Refused	**2.16 (1.51 to 3.09, p<0.001)**	1.30 (0.87 to 1.95, p=0.206)	**1.46 (1.02 to 2.10, p=0.040)**
Yes	**1.81 (1.38 to 2.39, p<0.001)**	1.01 (0.72 to 1.43, p=0.953)	1.08 (0.82 to 1.43, p=0.590)
Received intervention for alcohol cessation			
Not required	Ref	Ref	
No	1.06 (0.84 to 1.34, p=0.624)	0.91 (0.58 to 1.43, p=0.680)	
Refused	1.32 (1.03 to 1.69, p=0.029)	1.04 (0.73 to 1.48, p=0.842)	
Yes	1.20 (0.91 to 1.58, p=0.207)	0.86 (0.62 to 1.19, p=0.367)	
Received intervention for smoking cessation			
Not required	Ref	Ref	
No	**1.42 (1.13 to 1.80, p=0.003)**	0.94 (0.61 to 1.45, p=0.771)	
Refused	**1.82 (1.52 to 2.19, p<0.001)**	1.24 (0.96 to 1.60, p=0.106)	
Yes	**1.61 (1.36 to 1.90, p<0.001)**	1.16 (0.94 to 1.44, p=0.157)	
Received intervention for substance use			
Not required	Ref	Ref	Ref
No	**1.49 (1.16 to 1.91, p=0.002)**	1.54 (0.93 to 2.54, p=0.090)	1.27 (0.96 to 1.68, p=0.100)
Refused	**2.45 (2.01 to 3.00, p<0.001)**	**1.50 (1.11 to 2.03, p=0.008)**	**1.71 (1.38 to 2.13, p<0.001)**
Yes	**2.55 (2.17 to 3.00, p<0.001)**	**1.48 (1.20 to 1.83, p<0.001)**	**1.48 (1.23 to 1.78, p<0.001)**
Received intervention for weight loss			
Not required	Ref	Ref	
No	0.91 (0.61 to 1.34, p=0.625	0.71 (0.41 to 1.22, p=0.212)	
Refused	1.07 (0.73 to 1.55, p=0.730)	1.07 (0.66 to 1.76, p=0.776)	
Yes	0.87 (0.75 to 1.01, p=0.063)	1.08 (0.91 to 1.30, p=0.380)	

* For the continuous variables ‘care coordinator caseload’ and ‘proportion meeting waiting time standard’, stated hazard ratios indicate the change in hazard with a one-unit increase in the exposure. For example, in the unadjusted model each additional person on a care coordinator’s caseload increased the hazard of relapse by 1% (HR 1.01, 95% CI 1.00 to 1.01, p=0.046)

## Data Availability

RW, EP and JA had access to the full study dataset. The dataset and code script for analysis is held in the Office for National Statistics’ Secure Research Service and as such it is unfortunately not possible to share on request. Statistical data from the ONS is subject to Crown Copyright. The use of the ONS statistical data in this work does not imply the endorsement of the ONS in relation to the interpretation or analysis of the statistical data. This work uses research datasets which may not exactly reproduce National Statistics aggregates.
