# Supplementary material for: From Early Intervention in Psychosis to Intensive Care: correlates of restrictive psychiatric practice in a national retrospective cohort study
Source: Br J Psychiatry. Author manuscript; Available in PMC 2026 Jun 15. (PMC7619183; doi:10.1192/bjp.2026.10637)
Supplement: Supplementary Material [file EMS213054-supplement-Supplementary_Material.pdf]

FROM EARLY INTERVENTION IN PSYCHOSIS TO INTENSIVE CARE: CORRELATES OF RESTRICTIVE PSYCHIATRIC PRACTICE IN A NATIONAL RETROSPECTIVE COHORT STUDY

Ryan Williams, Ed Penington, Veenu Gupta, Michelle Rickett, Joel Agorinya, Apostolos Tsiachristas, Carolyn Chew-Graham, Jonathan Woodward, David Shiers, Alex Bottle, Benjamin McNeillis, Paul French, Belinda Lennox, Mike J Crawford

Supplementary Appendix

CONTENTS

STROBE CHECKLIST....3
NCAP COHORT – LINKAGE WITH MHSDS....6
DETAILED STATISTICAL METHODS....8
COHORT DEMOGRAPHICS ....**Error! Bookmark not defined.**
TABLE A1....**Error! Bookmark not defined.**
FIGURE A1 – Detailed breakdown of ethnicity 10
ANALYSIS – SECONDARY OUTCOMES....12
TABLE A2 - SECLUSION (those admitted to PICU only)....12
TABLE A3 - PHYSICAL RESTRAINT (for those who were admitted to PICU or general acute wards).14
TABLE A4 - INJECTED CHEMICAL RESTRAINT (for those who were admitted to PICU or general acute wards)....16
TABLE A5 - REQUESTS FOR POLICE ASSISTANCE (of those who were admitted to PICU or general acute wards)....18
REFERENCES (Appendix) 23

STROBE CHECKLIST

STROBE Statement[1] —Checklist of items that should be included in reports of ***cohort studies***

	Item No	Recommendation	Reported on page
**Title and abstract**	1	(*a*) Indicate the study's design with a commonly used term in the title or the abstract	p.1
		(*b*) Provide in the abstract an informative and balanced summary of what was done and what was found	p.2
**Introduction**			
Background/rationale	2	Explain the scientific background and rationale for the investigation being reported	p.3
Objectives	3	State specific objectives, including any prespecified hypotheses	p.3
**Methods**			
Study design	4	Present key elements of study design early in the paper	p.4-5
Setting	5	Describe the setting, locations, and relevant dates, including periods of recruitment, exposure, follow-up, and data collection	p.4
Participants	6	(*a*) Give the eligibility criteria, and the sources and methods of selection of participants. Describe methods of follow-up	p.4
		(*b*) For matched studies, give matching criteria and number of exposed and unexposed	N/A
Variables	7	Clearly define all outcomes, exposures, predictors, potential confounders, and effect modifiers. Give diagnostic criteria, if applicable	p.5
Data sources/ measurement	8*	For each variable of interest, give sources of data and details of methods of assessment (measurement). Describe comparability of assessment methods if there is more than one group	p.5
Bias	9	Describe any efforts to address potential sources of bias	p.4-5

Study size	10	Explain how the study size was arrived at	p.4
Quantitative variables	11	Explain how quantitative variables were handled in the analyses. If applicable, describe which groupings were chosen and why	p.4-5, Appendix p.7-8
Statistical methods	12	(*a*) Describe all statistical methods, including those used to control for confounding	p.5, Appendix p.8
		(*b*) Describe any methods used to examine subgroups and interactions	N/A
		(*c*) Explain how missing data were addressed	p.5
		(*d*) If applicable, explain how loss to follow-up was addressed	N/A
		(*e*) Describe any sensitivity analyses	p.5
**Results**			
Participants	13*	(a) Report numbers of individuals at each stage of study—e.g. numbers potentially eligible, examined for eligibility, confirmed eligible, included in the study, completing follow-up, and analysed	p.7
		(b) Give reasons for non-participation at each stage	N/A
		(c) Consider use of a flow diagram	N/A
Descriptive data	14*	(a) Give characteristics of study participants (e.g. demographic, clinical, social) and information on exposures and potential confounders	p.16-17
		(b) Summarise follow-up time (e.g. average and total amount)	p.4
Outcome data	15*	Report numbers of outcome events or summary measures over time	p.16-17
Main results	16	(*a*) Give unadjusted estimates and, if applicable, confounder-adjusted estimates and their precision (e.g. 95% confidence interval). Make clear which confounders were adjusted for and why they were included	p.18-19, Appendix p.11-22
		(*b*) Report category boundaries when continuous variables were categorized	N/A

		(*c*) If relevant, consider translating estimates of relative risk into absolute risk for a meaningful time period	Appendix p.19
Other analyses	17	Report other analyses done—e.g. analyses of subgroups and interactions, and sensitivity analyses	p.8<br><br>Appendix p.11-22
**Discussion**			
Key results	18	Summarise key results with reference to study objectives	p.9-11
Limitations	19	Discuss limitations of the study, taking into account sources of potential bias or imprecision. Discuss both direction and magnitude of any potential bias	p.10
Interpretation	20	Give a cautious overall interpretation of results considering objectives, limitations, multiplicity of analyses, results from similar studies, and other relevant evidence	p.10-11
Generalisability	21	Discuss the generalisability (external validity) of the study results	p.10
**Other information**			
Funding	22	Give the source of funding and the role of the funders for the present study and, if applicable, for the original study on which the present article is based	p.12

*Give information separately for exposed and unexposed groups.

Note: An Explanation and Elaboration article discusses each checklist item and gives methodological background and published examples of transparent reporting. The STROBE checklist is best used in conjunction with this article (freely available on the Web sites of PLoS Medicine at http://www.plosmedicine.org/, Annals of Internal Medicine at http://www.annals.org/, and Epidemiology at http://www.epidem.com/). Information on the STROBE Initiative is available at http://www.strobe-statement.org.

NCAP COHORT – LINKAGE WITH MHSDS

The following section provides detailed information on deterministic linkage between NCAP and MHSDS records undertaken by NHS Digital, cohort de-duplication across audit rounds, and data cleaning decisions. A summary of these procedures is provided in the main manuscript.

The NCAP is a yearly case note audit of representative sample of the caseload of all EIP services in England at specified census dates. For all teams, a sample of 100 individuals is selected (or all eligible service users if there are fewer than 100) who have been on the caseload for at least 6 months. The case notes of these service users are then used to answer a questionnaire, which collects data on service user characteristics and receipt of EIP components. Data on receipt of EIP components were obtained from the National Clinical Audits of Psychosis conducted in 2019/20 and 2020/21, with audit census dates of 1st April 2019 and 1st April 2020 respectively. The full cohort is taken from the full set of individuals included in the 2019/20 and 2020/21 National Clinical Audits of Psychosis (NCAP) whose personal identifying information was submitted to NHS Digital for linking with other NHS records. No additional cleaning of NCAP records was necessary as these have been cleaned for analysis by RCPsych.

Each NCAP record is uniquely identified by a unique identifying variable submitted alongside the cohort. No equivalent unique identifier is available for the NHS data, so duplicate NCAP records (e.g. someone included in both the 2019/20 and 2020/21 NCAP) were identified by merging with the list of referrals to mental health services (MHSDS table MHS101). If two NCAP records linked to the same uniquely identified referral, they are assumed to have been linked to the same master patient identifier by NHS Digital producing a set of Unique IDs across NCAP rounds. A small number of de-duplicated cohort records link the same set of NHS Digital records to more than one NCAP record within a given year, or round of the NCAP. For example, the same individual might appear in two separate EIP teams in 2019/20 and appear once in 2020/21. Manual review of the NCAP records in question (e.g. age, gender, ethnicity) suggests that these are not the same individuals on the books of multiple EIP teams, but have been incorrectly matched with NHS Digital records as part of the linking process. All of these observations have been excluded, rather than attempting to identify the correct NCAP record for each case. It should also be noted that this raises questions about the accuracy of the linking process in general: only a very small number of NCAP records have been assigned to overlapping NHS identifiers, but this is from an extremely large population of potential NHS identifiers.

Table A1: NCAP linked cohort cleaning

	Overall (N=20593)
**Linked to any MHS101 referral**	
0	2294 (11.1%)
1	18299 (88.9%)
**Unique ID across NCAP rounds**	
0	3400 (18.6%)
1	14899 (81.4%)
Missing	2294 (11.1%)
**Unique ID within NCAP rounds**	
0	25 (0.2%)
1	14874 (99.8%)
Missing	5694 (27.7%)

	Overall (N=20593)

Initial population consists of all individual records from the 2019/20 and 2020/21 National Clinical Audits of Psychosis (NCAPs). Each subsequent population is the remaining records after those not meeting the cleaning criteria have been removed.

PRIMARY DATA SOURCE FOR EACH VARIABLE

For fixed or quasi-fixed individual characteristics – age (derived from year of birth), sex and ethnicity - NCAP was treated as the primary data source. NCAP data are derived from a structured audit of patient case notes undertaken by local clinical teams, and demonstrate greater completeness and internal consistency for these variables than administrative records. For variables relating to components of EIP care, NCAP was also treated as authoritative, as these variables were explicitly defined.

MHSDS was the primary data source for outcomes only (PICU admission, seclusion, restraint and police involvement).

DISCORDANT DATA HANDLING

In linked routine data studies, the same variables may be recorded in more than one data source. In this study, demographic characteristics and some variables relating to components of care were available from both the NCAP and MHSDS, whereas outcome variables relating to restrictive interventions were available only from MHSDS.

Discordance within NCAP across audit rounds

A minority of individuals appeared in more than one NCAP audit round (2019/20 and 2020/21). Discordant values for fixed demographic characteristics across NCAP rounds were uncommon. For example, inconsistencies in year of birth were observed in approximately 0.4% of individuals with records in both rounds, and inconsistencies in sex were observed in approximately 0.2%. Where discordant values were observed across NCAP rounds, the most recent NCAP record was used.

Discordance between NCAP and MHSDS

Demographic variables were also available in MHSDS; however, comparison of NCAP and MHSDS records demonstrated substantially greater internal inconsistency within MHSDS for age-derived variables. Date of birth was not available (as this had been removed as a potential identifier) but 36% of individuals had multiple, conflicting 'age at referral' values recorded across different MHSDS referral records, often differing by one year but occasionally by larger margins, likely reflecting repeated data entry and administrative updating over time.

Given these patterns, no further attempt was made to reconcile NCAP demographic variables using MHSDS data. Instead, NCAP values were retained as the primary source for demographic characteristics, and MHSDS-derived demographic variables were not used in the analysis. This approach was chosen to minimise measurement error in fixed covariates and to avoid introducing additional uncertainty through post hoc reconciliation of administrative records.

Summary of approach

In summary, NCAP was treated as the authoritative source for demographic and EIP care variables, while MHSDS was used for outcomes. Discordant demographic values across NCAP rounds were rare and resolved by using the most recent audit record. Discordance between NCAP and MHSDS demographic data was not resolved by harmonisation, due to greater inconsistency in administrative records; instead, NCAP data were prioritised throughout.

DETAILED STATISTICAL METHODS

All analyses were performed using 'R'.[2] We conducted a comprehensive analysis of the associations between exposure variables and primary and secondary outcomes while accounting for potential confounding factors.

Initially, we generated descriptive statistics for all exposure variables, outcome measures and covariates. We used unadjusted tests to explore relationships between the exposure variables, covariates and primary and secondary outcomes.

Next, for each outcome we fit an adjusted (multivariable) regression model including all exposures and covariates identified as potentially significant from our a priori planning – to consider the simultaneous (rather than isolated) effect of all variables, taking potential confounding effects into account. We used multilevel regression models to account for clustering effects (as patients in the cohort are grouped within EIP services). We included a random intercept for EIP team to account for potential team-level variations, and random slopes between EIP team and NCAP audit round to account for yearly differences e.g. due to COVID-19. These were included to account for clustering by audit round and to estimate associations averaged across the study period, rather than to estimate year or team-specific effects. This approach acknowledges the potential correlation between individuals within the same service, ensuring appropriate adjustments were made to obtain unbiased estimates. It also allows service-level effects to be handled properly.[3]

Finally, we built refined statistical models for each analysis on the basis of the following pragmatic principles:

- Explanatory variables hypothesised to be associated with outcomes from a-priori discussions and previous studies were accounted for in model exploration;
- Demographic variables were included in model exploration;
- Final model selection was performed using a 'criterion-based' approach, aiming to minimise the Akaike Information Criterion (AIC) to establish models that captured as much information in the data with as few parameters as possible.[4]

Given the smaller number of events for some secondary outcomes, retaining all candidate covariates risked overfitting; therefore, final models were selected to balance interpretability and model fit.

We examined associations between exposures and outcomes) using Cox regression. Cox regression allows for the analysis of time-to-event data while accommodating censoring effects, which occurred if participants had not experienced the event of interest during the study period. The time of exposure was taken as date of the relevant NCAP audit period for each individual (1st April 2019 or 1st April 2020).

We assessed model assumptions for Cox regression models (proportional hazards) using Schoenfeld residuals, both statistically via the *cox.zph* function in R (global Grambsch–Therneau test) and visually through plots of residuals over time. Multicollinearity was checked using variance inflation factors (VIF) for all covariates. Unfortunately, plots of residuals were considered potentially identifiable data and were not cleared for extraction from the ONS secure research service environment.

Prior to study initiation, we estimated that the available sample size would provide adequate statistical power to detect small differences in the likelihood of the primary outcome across exposures and to yield acceptably precise regression coefficient estimates, based on the widely-used standard of 20 outcomes per exposure variable, derived from previous studies.[5]

TABLE A1 – Source of exposure and outcome variables

Variable	Data source	Derivation
**PATIENT-LEVEL EXPOSURES**		
CBT for psychosis	NCAP	Recorded based on documentation in clinical case notes.
Family intervention	NCAP	Recorded based on documentation in clinical case notes.
Vocational intervention	NCAP	Recorded based on documentation in clinical case notes.
Clozapine	NCAP	Recorded based on documentation in clinical case notes. Eligibility recorded using NCAP audit item specifying two adequate but unsuccessful trials of antipsychotic medication.
Carer-focused intervention	NCAP	Recorded based on documentation in clinical case notes
Smoking intervention	NCAP	Recorded based on documentation in clinical case notes.
Substance use intervention	NCAP	Recorded based on documentation in clinical case notes.
Alcohol use intervention	NCAP	Recorded based on documentation in clinical case notes.
Intervention for weight loss	NCAP	Recorded based on documentation in clinical case notes.
**SERVICE-LEVEL EXPOSURES**		
Mean care coordinator caseload at treating service	NCAP (contextual questionnaire)	Derived from service-level contextual questionnaire completed by EIP teams, reporting mean caseload per care coordinator
Proportion of caseload meeting 2-week waiting time standard at treating service	NCAP (contextual questionnaire)	Derived from service-level contextual questionnaire reporting the proportion of patients meeting the NICE 2-week waiting time standard
**OUTCOMES**		
PICU admission	MHSDS	Identified using ward type and transfer codes within inpatient spell records (MHS501)
Seclusion	MHSDS	Recorded as a seclusion event in the MHSDS restrictive interventions table (MHS505)
Physical restraint	MHSDS	Recorded as physical restraint in the MHSDS restrictive interventions table (MHS505)
Injected chemical restraint	MHSDS	Recorded as injected rapid tranquillisation in the MHSDS restrictive interventions table (MHS505)
Request for police assistance	MHSDS	Identified via requested police attendance table (MHS516)

FIGURE A1 – Detailed breakdown of ethnicity

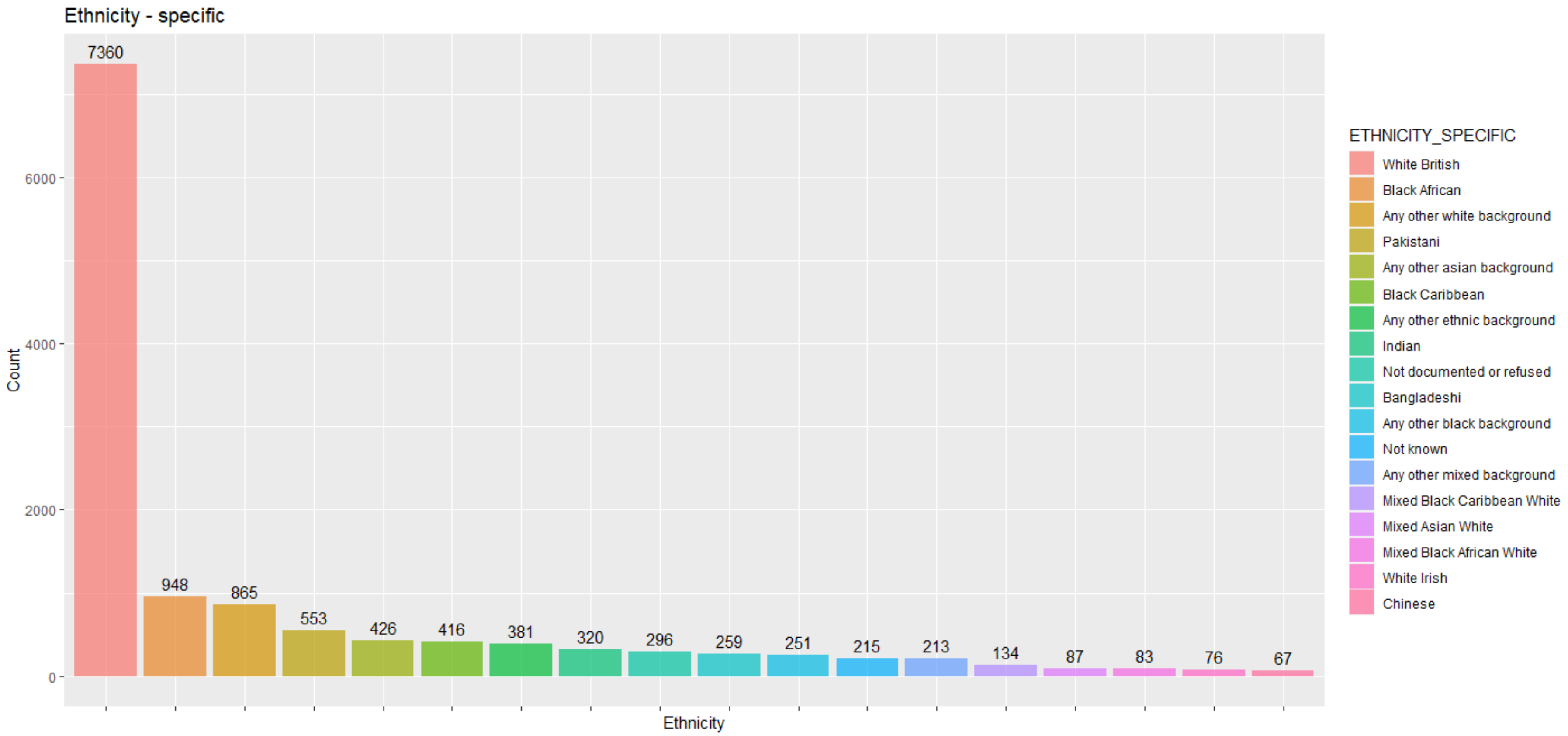

210

ANALYSIS – SECONDARY OUTCOMES

TABLE A2 - SECLUSION (those admitted to PICU only)

This table presents the unadjusted and adjusted hazard ratios (HRs) with 95% confidence intervals (CIs) for the primary outcome (PICU admission). The 'Full Model' includes all exposure variables and covariates, while the 'Final Model' is based on a refined selection of variables informed by statistical and theoretical considerations. Hazard ratios represent the relative likelihood of seclusion occurring at any given time for individuals in one category of a variable compared with the reference category, holding all other variables constant. HR > 1 indicates an increased likelihood of seclusion, while HR < 1 indicates a decreased likelihood. Results are adjusted for clustering within services. Results in **bold** indicate p-values ≤0.05.

Variables	Unadjusted HR (95% CI)	Adjusted HR - Full Model (95% CI)	Adjusted HR - Final Model (95% CI)
Age			
<25	1.35 (0.92 to 2.00, p=0.129)	1.38 (0.86 to 2.20, p=0.181)	1.38 (0.86 to 2.20, p=0.181)
25-34	Ref	Ref	Ref
35-44	**0.79 (0.60 to 0.95, p=0.048)**	**0.83 (0.57 to 0.95, p=0.045)**	**0.80 (0.56 to 0.98, p=0.048)**
45-54	**0.34 (0.05 to 0.84, p=0.028)**	**0.34 (0.05 to 0.88, p=0.029)**	0.34 (0.10 to 1.02, p=0.053)
>54	**0.00 (0.00 to Inf, p=0.993)**	0.00 (0.00 to Inf, p=0.998)	0.00 (0.00 to Inf, p=0.998)
Sex			
Female (or other)	Ref	Ref	
Male	1.45 (0.85 to 2.46, p=0.170)	1.08 (0.62 to 1.90, p=0.785)	
Ethnicity			
White	Ref	Ref	
Black/ Black British	1.49 (0.95 to 2.32, p=0.080)	1.48 (0.97 to 3.08, p=0.053)	
Asian/ Asian British	1.27 (0.71 to 2.30, p=0.424)	0.98 (0.46 to 2.08, p=0.960)	
Mixed	1.28 (0.63 to 2.59, p=0.499)	1.02 (0.45 to 2.29, p=0.964)	
Other	0.41 (0.10 to 1.68, p=0.216)	0.43 (0.10 to 1.83, p=0.255)	
Refused	0.00 (0.00 to Inf, p=0.993)	0.00 (0.00 to Inf, p=0.996)	
Unknown/ Undocumented	1.82 (0.66 to 5.04, p=0.247)	1.95 (0.57 to 6.72, p=0.290)	
Patient in employment or education			
No	Ref	Ref	
Yes	0.94 (0.64 to 1.38, p=0.750)	1.03 (0.68 to 1.58, p=0.874)	
PICU admission prior to EIP involvement			
No	Ref	Ref	
Yes	1.32 (0.91 to 1.91, p=0.147)	0.99 (0.63 to 1.56, p=0.972)	
Number of non-PICU admissions following EIP involvement			
0	Ref	Ref	
1-2	1.32 (0.83 to 2.08, p=0.237)	1.20 (0.74 to 1.95, p=0.460)	
3+	0.88 (0.47 to 1.65, p=0.687)	0.80 (0.41 to 1.55, p=0.501)	
Average care coordinator caseload at treating EIP service*			
	1.02 (0.99 to 1.05, p=0.263)	1.02 (0.98 to 1.05, p=0.327)	
Likelihood that treatment began in <2 weeks (based on proportion meeting waiting time standard at treating service)*			
	**1.01 (1.00 to 1.03, p=0.026)**	1.01 (0.99 to 1.03, p=0.074)	
Received Cognitive Behavioural Therapy for Psychosis			
No	Ref	Ref	
Refused	0.95 (0.57 to 1.59, p=0.850)	0.77 (0.43 to 1.40, p=0.390)	
Yes	1.00 (0.62 to 1.63, p=0.991)	0.79 (0.45 to 1.37, p=0.394)	
Received Family Intervention			
No	Ref	Ref	
Refused	0.97 (0.63 to 1.51, p=0.904)	0.97 (0.58 to 1.62, p=0.912)	
Yes	1.17 (0.73 to 1.87, p=0.505)	1.00 (0.58 to 1.73, p=0.997)	
Received carer-focussed intervention			
Not eligible	Ref	Ref	
No	0.64 (0.38 to 1.09, p=0.102)	0.66 (0.37 to 1.17, p=0.512)	
Yes	1.06 (0.67 to 1.68, p=0.798)	1.04 (0.61 to 1.79, p=0.873)	
Received vocational support			
No	Ref	Ref	
Refused	0.95 (0.59 to 1.52, 0=0.826)	1.39 (0.81 to 2.37, p=0.228)	
Yes	0.96 (0.62 to 1.49, p=0.864)	1.08 (0.67 to 1.76, p=0.743)	

Received clozapine			
Not eligible	Ref	Ref	Ref
Not offered	**1.72 (1.01 to 2.95, p=0.046)**	1.65 (0.98 to 2.94, p=0.051)	1.65 (0.98 to 2.83, p=0.052)
Refused	1.70 (0.94 to 3.90, p=0.059)	**1.63 (0.99 to 3.18, p=0.050)**	1.62 (0.97 to 3.71, p=0.056)
Yes	1.46 (0.73 to .91, p=0.280)	1.27 (0.60 to 2.70, p=0.523)	1.26 (0.68 to 2.71, p=0.383)
Received intervention for alcohol cessation			
Not required	Ref	Ref	
No	0.82 (0.41 to 1.62, p=0.560)	2.45 (0.98 to 6.15, p=0.056)	
Refused	0.60 (0.26 to 1.37, p=0.226)	0.47 (0.16 to 1.37, p=0.168)	
Yes	1.23 (0.62 to 2.43, p=0.562)	0.94 (0.46 to 1.95, p=0.873)	
Received intervention for smoking cessation			
Not required	Ref	Ref	
No	0.53 (0.22 to 1.24, p=0.143	0.55 (0.16 to 1.86, p=0.334)	
Refused	1.04 (0.62 to 1.74, p=0.889	1.27 (0.69 to 2.33, p=0.445)	
Yes	**1.55 (1.01 to 2.39, p=0.047)**	1.42 (0.86 to 2.34, p=0.174)	
Received intervention for substance use			
Not required	Ref	Ref	
No	0.48 (0.19 to 1.20, p=0.118)	0.48 (0.12 to 1.91, p=0.295)	
Refused	0.81 (0.45 to 1.45, p=0.471)	0.99 (0.48 to 2.06, p=0.984)	
Yes	1.24 (0.82 to 1.88, p=0.305)	1.02 (0.63 to 1.66, p=0.931)	
Received intervention for weight loss			
Not required	Ref	Ref	
No	0.58 (0.14 to 2.37, p=0.449)	0.58 (0.14 to 2.45, p=0.457)	
Refused	2.04 (0.94 to 4.45, p=0.072)	2.27 (0.99 to 5.23, p=0.054)	
Yes	0.99 (0.66 to 1.50, p=0.981)	0.78 (0.50 to 1.21, p=0.263)	

TABLE A3 - PHYSICAL RESTRAINT (for those who were admitted to PICU or general acute wards)

This table presents the unadjusted and adjusted hazard ratios (HRs) with 95% confidence intervals (CIs) for the primary outcome (PICU admission). The ‘Full Model’ includes all exposure variables and covariates, while the ‘Final Model’ is based on a refined selection of variables informed by statistical and theoretical considerations. Hazard ratios represent the relative likelihood of physical restraint occurring at any given time for individuals in one category of a variable compared with the reference category, holding all other variables constant. HR > 1 indicates an increased likelihood of physical restraint, while HR < 1 indicates a decreased likelihood. Results are adjusted for clustering within services. Results in **bold** indicate p-values ≤0.05.

Variables	Unadjusted HR (95% CI)	Adjusted HR - Full Model (95% CI)	Adjusted HR - Final Model (95% CI)
Age			
<25	**1.49 (1.18 to 1.87, p=0.001)**	**1.35 (1.04 to 1.75, p=0.026)**	**1.44 (1.12 to 1.83, p=0.004)**
25-34	Ref	Ref	Ref
35-44	**0.63 (0.44 to 0.90, p=0.012)**	0.78 (0.53 to 1.16, p=0.220)	0.73 (0.50 to 1.06, p=0.099)
45-54	0.58 (0.32 to 1.04, p=0.066)	0.86 (0.46 to 1.63, p=0.652)	0.79 (0.42 to 1.46, p=0.445)
>54	**0.25 (0.08 to 0.77, p=0.016)**	0.47 (0.15 to 1.50, p=0.203)	0.40 (0.13 to 1.27, p=0.120)
Sex			
Female (or other)	Ref	Ref	Ref
Male	**3.20 (2.17 to 4.73, p<0.001)**	**1.88 (1.25 to 2.82, p=0.002)**	**2.05 (1.37 to 3.06, p<0.001)**
Ethnicity			
White	Ref	Ref	Ref
Black/ Black British	**1.46 (1.11 to 1.93, p=0.007)**	1.39 (0.95 to 1.71, p=0.105)	**1.42 (1.03 to 1.93, p=0.033)**
Asian/ Asian British	**1.55 (1.14 to 2.11, p=0.005)**	1.27 (1.00 to 1.93, p=0.052)	1.37 (0.99 to 1.98, p=0.060)
Mixed	1.00 (0.59 to 1.69, p=0.995)	0.72 (0.42 to 1.25, p=0.244)	0.72 (0.40 to 1.31, p=0.284)
Other	1.25 (0.68 to 2.31, p=0.468)	0.83 (0.44 to 1.59, p=0.579)	1.12 (0.58 to 2.14, p=0.738)
Refused	1.48 (0.78 to 2.81, p=0.225)	0.89 (0.41 to 1.89, p=0.753)	0.97 (0.44 to 2.10, p=0.929)
Unknown/ Undocumented	1.71 (0.80 to 3.63, p=0.166)	1.28 (0.56 to 2.89, p=0.559)	1.17 (0.47 to 2.87, p=0.739)
Patient in employment or education			
No	Ref	Ref	
Yes	0.87 (0.65 to 1.18, p=0.369)	0.80 (0.58 to 1.11, p=0.179)	
PICU admission pre EIP involvement			
No	Ref	Ref	
Yes	**2.10 (1.66 to 2.65, p<0.001)**	1.16 (0.87 to 1.54, p=0.303)	
PICU admission post EIP involvement			
No	Ref	Ref	Ref
Yes	**4.70 (3.80 to 5.82, p<0.001)**	**5.03 (3.85 to 6.57, p<0.001)**	**4.59 (3.57 to 5.91, p<0.001)**
Number of non-PICU admissions following EIP involvement			
0	Ref	Ref	Ref
1-2	**0.44 (0.29 to 0.56, p<0.001)**	1.37 (0.93 to 2.02, p=0.110)	1.42 (0.98 to 2.05, p=0.064)
3+	0.73 (0.50 to 1.07, p=0.103)	**1.84 (1.22 to 2.79, p=0.004)**	**1.84 (1.24 to 2.72, p=0.003)**
Average care coordinator caseload at treating EIP service*			
	0.99 (0.97 to 1.02, p=0.523)	0.98 (0.95 to 1.01, p=0.146)	
Likelihood that treatment began in <2 weeks (based on proportion meeting waiting time standard at treating service)*			
	0.99 (0.98 to 1.00, p=0.193)	1.00 (0.99-1.01, p=0.869)	
Received Cognitive Behavioural Therapy for Psychosis			
No	Ref	Ref	Ref
Refused	0.91 (0.62 to 1.32, p=0.603)	0.82 (0.54 to 1.25, p=0.355)	0.91 (0.62 to 1.32, p=0.609)
Yes	**0.54 (0.38 to 0.77, p=0.001)**	**0.59 (0.40 to 0.87, p=0.009)**	**0.68 (0.47 to 0.98, p=0.037)**
Received Family Intervention			
No	Ref	Ref	
Refused	1.04 (0.74 to 1.47, p=0.804)	1.00 (0.68 to 1.47, p=0.991)	
Yes	1.23 (0.86 to 1.76, p=0.261)	1.18 (0.78 to 1.78, p=0.429)	
Received carer-focussed intervention			
Not eligible	Ref	Ref	
No	1.51 (1.01 to 2.24, p=0.638)	1.34 (0.88 to 2.04, p=0.657)	
Yes	**1.40 (0.96 to 2.06, p=0.010)**	1.13 (0.74 to 1.73, p=0.069)	
Received vocational support			
No	Ref	Ref	
Refused	1.10 (0.75 to 1.61, p=0.638)	1.10 (0.72 to 1.67, p=0.657)	
Yes	**1.56 (1.11 to 2.18, p=0.010)**	1.40 (0.97 to 2.02, p=0.069)	
Received clozapine			

Not eligible	Ref	Ref	Ref
Not offered	**2.58 (1.68 to 3.98, p<0.001)**	**2.33 (1.48 to 3.67, p<0.001)**	**2.39 (1.55 to 3.69, p<0.001)**
Refused	**2.20 (1.03 to 4.69, p=0.042)**	1.64 (0.76 to 3.53, p=0.205)	1.62 (0.76 to 3.46, p=0.215)
Yes	1.69 (0.91 to 3.11, p=0.095)	1.41 (0.76 to 2.63, p=0.281)	1.33 (0.72 to 2.46, p=0.365)
Received intervention for alcohol cessation			
Not required	Ref	Ref	
No	1.15 (0.71 to 1.86, p=0.568)	1.15 (0.55 to 2.44, p=0.707)	
Refused	0.94 (0.51 to 1.74, p=0.845)	0.61 (0.29 to 1.28, p=0.191)	
Yes	1.61 (0.96 to 2.69, p=0.073)	1.20 (0.69 to 2.10, p=0.511)	
Received intervention for smoking cessation			
Not required	Ref	Ref	
No	1.53 (0.95 to 2.48, p=0.081)	1.21 (0.60 to 2.45, p=0.600)	
Refused	**1.60 (1.07 to 2.30, p=0.023)**	1.22 (0.74 to 2.02, p=0.428)	
Yes	**1.69 (1.20 to 2.40, p=0.003)**	1.15 (0.77 to 1.72, p=0.501)	
Received intervention for substance use			
Not required	Ref	Ref	
No	1.31 (0.74 to 2.30, p=0.350)	0.68 (0.28 to 1.69, p=0.409)	
Refused	1.53 (0.98 to 2.39, p=0.061)	1.35 (0.78 to 2.35, p=0.288)	
Yes	**1.79 (1.29 to 2.49, p=0.001)**	1.37 (0.93 to 2.01, p=0.111)	
Received intervention for weight loss			
Not required	Ref	Ref	
No	0.73 (0.30 to 1.79, p=0.498)	0.84 (0.31 to 2.30, p=0.740)	
Refused	1.07 (0.50 to 2.30, p=0.853)	1.57 (0.72 to 3.42, p=0.256)	
Yes	**0.70 (0.50 to 0.96, p=0.029)**	0.81 (0.57 to 1.15, p=0.231)	

TABLE A4 - INJECTED CHEMICAL RESTRAINT (for those who were admitted to PICU or general acute wards)

This table presents the unadjusted and adjusted hazard ratios (HRs) with 95% confidence intervals (CIs) for the primary outcome (PICU admission). The 'Full Model' includes all exposure variables and covariates, while the 'Final Model' is based on a refined selection of variables informed by statistical and theoretical considerations. Hazard ratios represent the relative likelihood of injected chemical restraint occurring at any given time for individuals in one category of a variable compared with the reference category, holding all other variables constant. HR > 1 indicates an increased likelihood of injected chemical restraint, while HR < 1 indicates a decreased likelihood. Results are adjusted for clustering within services. Results in **bold** indicate p-values ≤0.05.

Variables	Unadjusted HR (95% CI)	Adjusted HR - Full Model (95% CI)	Adjusted HR - Final Model (95% CI)
Age			
<25	**1.47 (1.16 to 1.85, p=0.001)**	**1.31 (1.00 to 1.70, p=0.047)**	**1.30 (1.09 to 1.77, p=0.008)**
25-34	Ref	Ref	Ref
35-44	**0.63 (0.44 to 0.90, p=0.011)**	0.77 (0.52 to 1.13, p=0.185)	0.66 (0.50 to 1.06, p=0.096)
45-54	0.57 (0.32 to 1.03, p=0.063)	0.84 (0.45 to 1.58, p=0.592)	0.71 (0.42 to 1.41, p=0.426)
>54	0.25 (0.08 to 0.77, p=0.016)	0.46 (0.14 to 1.47, p=0.190)	0.32 (0.13 to 1.22, p=0.118)
Sex			
Female (or other)	Ref	Ref	
Male	**1.75 (1.00 to 3.06, p=0.048)**	1.28 (0.70 to 2.32, p=0.425)	
Ethnicity			
White	Ref	Ref	Ref
Black/ Black British	1.45 (0.98 to 2.30, p=0.051)	1.23 (0.95 to 1.91, p=0.419)	1.30 (0.96 to 2.12, p=0.051)
Asian/ Asian British	1.38 (0.91 to 2.37, p=0.059)	1.21 (0.87 to 2.08, p=0.059)	1.28 (0.94 to 2.07, p=0.053)
Mixed	1.53 (0.73 to 3.20, p=0.610)	1.14 (0.52 to 2.53, p=0.742)	1.31 (0.88 to 2.56, p=0.066)
Other	1.30 (0.47 to 3.58, p=0.610)	0.79 (0.25 to 2.43, p=0.691)	1.02 (0.32 to 3.33, p=0.968)
Refused	**3.38 (1.62 to 7.07, p=0.001)**	**2.50 (1.07 to 5.85, p=0.035)**	**2.59 (1.06 to 6.30, p=0.036)**
Unknown/ Undocumented	1.34 (0.33 to 5.48, p=0.685)	1.19 (0.29 to 4.88, p=0.813)	1.43 (0.34 to 5.99, p=0.626)
Patient in employment or education			
No	Ref	Ref	
Yes	0.99 (0.61 to 1.61, p=0.971)	0.90 (0.53 to 1.52, p=0.702)	
PICU admission pre EIP involvement			
No	Ref	Ref	
Yes	**2.70 (1.66 to 3.45, p<0.001)**	1.03 (0.67 to 1.54, p=0.603)	
PICU admission post EIP involvement			
No	Ref	Ref	Ref
Yes	**4.72 (3.81 to 5.85, p<0.001)**	**5.03 (3.85 to 6.58, p<0.001)**	**4.12 (3.56 to 5.10, p<0.001)**
Number of non-PICU admissions following EIP involvement			
0	Ref	Ref	Ref
1-2	**0.60 (0.29 to 0.86, p<0.001)**	1.35 (0.91 to 1.98, p=0.133)	1.36 (0.98 to 1.87, p=0.064)
3+	0.69 (0.50 to 1.04, p=0.094)	**1.51 (1.20 to 2.14, p=0.005)**	**1.53 (1.24 to 2.12, p=0.003)**
Average care coordinator caseload at treating EIP service*			
	1.03 (0.99 to 1.07, p=0.130)	1.02 (0.98 to 1.06, o=0.283)	
Likelihood that treatment began in <2 weeks (based on proportion meeting waiting time standard at treating service)*			
	**0.98 (0.97 to 1.00, p=0.050)**	0.99 (0.97 to 1.01, p=0.182)	
Received Cognitive Behavioural Therapy for Psychosis			
No	Ref	Ref	Ref
Refused	0.81 (0.45 to 1.45, p=0.474)	0.86 (0.44 to 1.71, p=0.672)	0.88 (0.49 to 1.60, p=0.686)
Yes	**0.52 (0.29 to 0.93, p=0.029)**	0.64 (0.33 to 1.22, p=0.176)	0.57 (0.32 to 1.01, p=0.053)
Received Family Intervention			
No	Ref	Ref	
Refused	0.91 (0.53 to 1.56, p=0.728)	1.17 (0.63 to 2.17, p=0.626)	
Yes	0.82 (0.45 to 1.49, p=0.514)	1.17 (0.59 to 2.32, p=0.651)	
Received carer-focussed intervention			
Not eligible	Ref	Ref	
No	1.35 (0.73 to 2.50, p=0.337)	1.15 (0.60 to 2.19, p=0.671)	
Yes	0.90 (0.49 to 1.67, p=0.742)	0.76 (0.39 to 1.48, p=0.418)	
Received vocational support			
No	Ref	Ref	
Refused	0.60 (0.31 to 1.17, p=0.134)	0.65 (0.32 to 1.33, p=0.234)	
Yes	1.16 (0.69 to 1.96, p=0.566)	1.22 (0.69 to 2.15, p=0.497)	
Received clozapine			

Not eligible	Ref	Ref	
Not offered	1.76 (0.92 to 3.36, p=0.087)	1.81 (0.93 to 3.50, p=0.080)	
Refused	1.28 (0.40 to 4.08, p=0.681)	1.39 (0.43 to 4.52, p=0.583)	
Yes	0.25 (0.04 to 1.84, p=0.175)	0.29 (0.04 to 2.14, p=0.227)	
Received intervention for alcohol cessation			
Not required	Ref	Ref	
No	**1.93 (1.01 to 3.69, p=0.046)**	1.68 (0.79 to 4.63, p=0.333)	
Refused	0.22 (0.03 to 1.60, p=0.134)	0.15 (0.02 to 1.21, p=0.076)	
Yes	0.85 (0.31 to 2.34, p=0.751)	0.87 (0.30 to 2.50, p=0.795)	
Received intervention for smoking cessation			
Not required	Ref	Ref	
No	1.92 (0.93 to 3.98, p=0.080)	1.63 (0.56 to 4.76, p=0.367)	
Refused	1.43 (0.76 to 2.70, p=0.271)	1.80 (0.87 to 3.74, p=0.113)	
Yes	1.13(0.63 to 2.02, p=0.691)	1.13 (0.59 to 2.17, p=0.715)	
Received intervention for substance use			
Not required	Ref	Ref	
No	1.21 (0.51 to 2.86, p=0.661)	0.29 (0.07 to 1.13, p=0.074)	
Refused	0.91 (0.41 to 2.03, p=0.815)	0.96 (0.38 to 2.37, p=0.921)	
Yes	1.15 (0.66 to 2.01, p=0.613)	0.86 (0.46 to 1.63, p=0.653)	
Received intervention for weight loss			
Not required	Ref	Ref	
No	0.85 (0.50 to 1.46, p=0.557)	0.89 (0.46 to 1.69, p=0.718)	
Refused	1.04 (0.48 to 2.25, p=0.920)	1.33 (0.55 to 3.24, p=0.532)	
Yes	0.71 (0.41 to 1.25, p=0.238)	0.72 (0.37 to 1.41, p=0.342)	

TABLE A5 - REQUESTS FOR POLICE ASSISTANCE (of those who were admitted to PICU or general acute wards)

This table presents the unadjusted and adjusted hazard ratios (HRs) with 95% confidence intervals (CIs) for the primary outcome (PICU admission). The ‘Full Model’ includes all exposure variables and covariates, while the ‘Final Model’ is based on a refined selection of variables informed by statistical and theoretical considerations. Hazard ratios represent the relative likelihood of a request for police assistance occurring at any given time for individuals in one category of a variable compared with the reference category, holding all other variables constant. HR > 1 indicates an increased likelihood of a request for police assistance, while HR < 1 indicates a decreased likelihood. Results are adjusted for clustering within services. Results in **bold** indicate p-values ≤0.05.

Variables	Unadjusted HR (95% CI)	Adjusted HR - Full Model (95% CI)	Adjusted HR - Final Model (95% CI)
Age			
<25	1.59 (0.96 to 2.63, p=0.069)	1.68 (0.97 to 2.90, p=0.063)	
25-34	Ref	Ref	
35-44	0.45 (0.20 to 1.02, p=0.056)	0.70 (0.28 to 1.73, p=0.435)	
45-54	0.36 (0.11 to 1.17, p=0.090)	1.11 (0.32 to 3.83, p=0.872)	
>54	0.00 (0.00 to Inf, p=0.994)	0.00 (0.00 to Inf, p=0.994)	
Sex			
Female (or other)	Ref	Ref	
Male	**2.37 (1.03 to 5.43, p=0.042)**	1.40 (0.55 to 4.61, p=0.482)	
Ethnicity			
White	Ref	Ref	Ref
Black/ Black British	1.81 (0.90 to 1.79, p=0.057)	1.16 (0.62 to 2.20, p=0.642)	1.26 (0.63 to 2.54, p=0.514)
Asian/ Asian British	0.87 (0.39 to 1.94, p=0.727)	0.95 (0.42 to 2.14, p=0.904)	0.87 (0.37 to 2.04, p=0.742)
Mixed	1.16 (0.42 to 3.25, p=0.773)	1.04 (0.37 to 2.94, p=0.934)	0.92 (0.31 to 2.71, p=0.878)
Other	1.50 (0.46 to 4.86, p=0.496)	1.20 (0.36 to 3.92, p=0.767)	1.81 (0.54 to 6.07, p=0.338)
Refused	0.00 (0.00 to Inf, p=0.995)	0.00 (0.00 to Inf, p=0.997)	0.00 (0.00 to Inf, p=0.995)
Unknown/ Undocumented	3.19 (0.99 to 10.34, p=0.053)	**3.35 (1.02 to 10.95, p=0.046)**	**3.86 (1.15 to 12.98, p=0.029)**
Patient in employment or education			
No	Ref	Ref	
Yes	0.63 (0.30 to 1.32, p=0.221)	0.84 (0.37 to 1.94, p=0.688)	
PICU admission pre EIP involvement			
No	Ref	Ref	Ref
Yes	**4.17 (2.50 to 6.94, p<0.001)**	1.53 (0.85 to 2.75, p=0.160)	
PICU admission post EIP involvement			
No	Ref	Ref	Ref
Yes	**15.77 (9.90 to 25.12, p<0.001)**	**4.32 (2.52 to 7.40, p<0.001)**	**5.90 (3.56 to 9.78, p<0.001)**
Number of non-PICU admissions following EIP involvement			
0	Ref	Ref	Ref
1-2	**7.94 (3.17 to 19.91, p<0.001)**	**8.70 (3.03 to 25.01, p<0.001)**	**6.70 (2.65 to 16.94, p<0.001)**
3+	**35.64 (14.03 to 90.54, p<0.001)**	**34.65 (11.61 to 103.41, p<0.001)**	**24.86 (9.52 to 64.94, p<0.001)**
Average care coordinator caseload at treating EIP service*			
	1.04 (0.98 to 1.27, p=0.119)	0.99 (0.92 to 1.08, p=0.583)	
Likelihood that treatment began in <2 weeks (based on proportion meeting waiting time standard at treating service)*			
	**0.97 (0.94 to 0.99, p=0.002)**	0.98 (0.95 to 1.01, p=0.080)	
Received Cognitive Behavioural Therapy for Psychosis			
No	Ref	Ref	
Refused	0.80 (0.32 to 1.96, p=0.623)	0.43 (0.15 to 1.24, p=0.118)	
Yes	0.59 (0.26 to 1.34, p=0.208)	0.60 (0.23 to 1.53, p=0.283)	
Received Family Intervention			
No	Ref	Ref	
Refused	1.38 (0.64 to 3.01, p=0.413)	0.91 (0.36 to 2.30, p=0.840)	
Yes	1.08 (0.43 to 2.67, p=0.876)	0.81 (0.29 to 2.26, p=0.684)	
Received carer-focussed intervention			
Not eligible	Ref	Ref	
No	1.22 (0.46 to 3.19, p=0.692)	0.92 (0.31 to 2.72, p=0.886)	
Yes	1.60 (0.66 to 3.85, p=0.297)	1.35 (0.51 to 3.58, p=0.543)	
Received vocational support			
No	Ref	Ref	
Refused	2.03 (0.83 to 4.98, p=0.120)	2.28 (0.76 to 6.88, p=0.142)	
Yes	1.95 (0.82 to 4.65, p=0.132)	2.12 (0.77 to 5.83, p-0.143)	
Received clozapine			

Not eligible	Ref	Ref	
Not offered	2.12 (0.75 to 6.04, p=0.158)	2.04 (0.68 to 6.11, p=0.201)	
Refused	0.00 (0.00 to Inf, p=1.00)	0.00 (0.00 to Inf, p=1.00)	
Yes	0.76 (0.10 to 5.56, p=0.785)	0.66 (0.09 to 5.02, p=0.689)	
Received intervention for alcohol cessation			
Not required	Ref	Ref	
No	1.48 (0.51 to 4.27, p=0.473)	0.71 (0.11 to 4.51, p=0.715)	
Refused	1.56 (0.47 to 5.21, p=0.466)	1.29 (0.27 to 6.11, p=0.744)	
Yes	2.44 (0.84 to 7.04, p=0.100)	2.03 (0.64 to 6.44, p=0.231)	
Received intervention for smoking cessation			
Not required	Ref	Ref	
No	2.19 (0.76 to 6.29, p=0.147)	0.66 (0.12 to 3.57, p=0.628)	
Refused	1.39 (0.48 to 4.00, p=0.542)	0.25 (0.05 to 1.16, p=0.077)	
Yes	**2.34 (1.05 to 5.21, p=0.038)**	1.01 (0.39 to 2.64, p=0.983)	
Received intervention for substance use			
Not required	Ref	Ref	Ref
No	**3.36 (1.18 to 9.52, p=0.023)**	**5.95 (1.03 to 34.34, p=0.046)**	**3.35 (1.16 to 9.69, p=0.026)**
Refused	**4.40 (1.73 to 11.19, p=0.002)**	**4.41 (1.25 to 15.50, p=0.021)**	**3.08 (1.18 to 8.05, p=0.022)**
Yes	**3.65 (1.58 to 8.44, p=0.003)**	1.77 (0.61 to 5.12, p=0.295)	1.96 (0.80 to 4.80, p=0.140)
Received intervention for weight loss			
Not required	Ref	Ref	
No	0.75 (0.10 to 5.56, p=0.780)	0.83 (0.19 to 6.23, p=0.889)	
Refused	0.78 (0.11 to 5.79, p=0.810)	1.01 (0.12 to 8.72, p=0.993)	
Yes	0.56 (0.25 to 1.25, p=0.156)	0.71 (0.28 to 1.78, p=0.460)	

TABLE A6 - Absolute time-to-event summaries for primary and secondary outcomes

Overall absolute time-to-event summaries for restrictive interventions and PICU admission. Absolute risks at two years were estimated using unstratified Kaplan–Meier methods. RMST denotes the restricted mean time free from the outcome up to two years of follow-up. These summaries are provided to contextualise the relative associations reported in multivariable models.

Outcome	N	Events, n	Event (%)	2-year absolute risk (%)	RMST to 2 years (days)
PICU admission	14,874	712	5.6	4.46	711.7
Seclusion	14,874	109	1.0	0.33	729.6
Physical restraint	14,874	336	2.3	0.78	729.2
Injected chemical restraint	14,874	125	0.8	0.32	729.7
Request for police assistance	14,874	68	0.5	0.12	729.9

SENSITIVITY ANALYSIS - clozapine-eligible patients only

TABLE A7 – PICU ADMISSION (clozapine eligible only)

This table presents the unadjusted and adjusted hazard ratios (HRs) with 95% confidence intervals (CIs) for the primary outcome (PICU admission) among only those patients eligible for clozapine (n=1908). The 'Full Model' includes all exposure variables and covariates, while the 'Final Model' is based on a refined selection of variables informed by statistical and theoretical considerations. Hazard ratios represent the relative likelihood of PICU admission occurring at any given time for individuals in one category of a variable compared with the reference category, holding all other variables constant. HR > 1 indicates an increased likelihood of PICU admission, while HR < 1 indicates a decreased likelihood. Results are adjusted for clustering within services. Results in **bold** indicate p-values ≤0.05.

Variables	Unadjusted HR (95% CI)	Adjusted HR - Full Model (95% CI)	Adjusted HR - Final Model (95% CI)
Age			
<25	**1.69 (1.13 to 2.52, p=0.011)**	**1.84 (1.15 to 2.95, p=0.011)**	**1.68 (1.11 to 2.56, p=0.015)**
25-34	Ref	Ref	Ref
35-44	**0.43 (0.21 to 0.86, p=0.017)**	0.55 (0.26 to 1.18, p=0.126)	**0.47 (0.23 to 0.95, p=0.036)**
45-54	0.28 (0.04 to 2.02, p=0.205)	0.36 (0.04 to 2.93, p=0.336)	0.31 (0.04 to 2.38, p=0.259)
>54	**0.00 (0.00 to 0.00, p<0.001)**	**0.00 (0.00 to 0.00, p<0.001)**	**0.00 (0.00 to 0.00, p<0.001)**
Sex			
Female (or other)	Ref	Ref	Ref
Male	**1.80 (1.11 to 2.92, p=0.018)**	1.34 (0.80 to 2.29, p=0.257)	1.30 (0.79 to 2.17, p=0.305)
Ethnicity			
White	Ref	Ref	Ref
Black/ Black British	**1.79 (1.14 to 2.82, p=0.012)**	**1.75 (1.03 to 2.94, p=0.039)**	**1.70 (1.07 to 2.70, p=0.026)**
Asian/ Asian British	0.67 (0.36 to 1.22, p=0.186)	0.73 (0.37 to 1.40, p=0.330)	0.65 (0.35 to 1.20, p=0.169)
Mixed	1.90 (0.71 to 3.15, p=0.284)	1.40 (0.67 to 2.88, p=0.373)	1.69 (0.52 to 2.29, p=0.820)
Other	**2.28 (1.03 to 5.06, p=0.043)**	1.98 (0.81 to 4.94, p=0.136)	**2.22 (1.01 to 4.89, p=0.047)**
Refused	0.45 (0.14 to 1.51, p=0.198)	0.33 (0.08 to 1.47, p=0.147)	0.40 (0.12 to 1.37, p=0.144)
Unknown/ Undocumented	**0.00 (0.00 to 0.00, p<0.001)**	**0.00 (0.00 to 0.00, p<0.001)**	**0.00 (0.00 to 0.00, p<0.001)**
Patient in employment or education			
No	Ref	Ref	
Yes	**0.80 (0.53 to 1.21, p=0.293)**	0.76 (0.45 to 1.07, p=0.097)	
PICU admission prior to EIP involvement			
No	Ref	Ref	Ref
Yes	**6.57 (4.53 to 9.52, p<0.001)**	**4.64 (2.85 to 7.57, p<0.001)**	**5.12 (3.35 to 7.82, p<0.001)**
Number of non-PICU admissions following EIP involvement			
0	Ref	Ref	Ref
1-2	**2.29 (1.43 to 3.65, p<0.001)**	**1.83 (1.14 to 2.95, p=0.013)**	**1.97 (1.24 to 3.14, p=0.004)**
3+	**4.34 (2.53 to 7.44, p<0.001)**	**2.67 (1.39 to 5.12, p=0.003)**	**3.37 (1.90 to 5.98, p<0.001)**
Average care coordinator caseload at treating EIP service*			
	1.00 (0.96 to 1.04, p=0.963)	1.00 (0.96 to 1.04, p=0.966)	
Likelihood that treatment began in <2 weeks (based on proportion meeting waiting time standard at treating service)*			
	0.99 (0.98 to 1.01, p=0.643)	1.00 (0.99 to 1.01, p=0.844)	
Received Cognitive Behavioural Therapy for Psychosis			
No	Ref	Ref	
Refused	1.22 (0.72 to 2.05, p=0.466)	1.72 (0.91 to 3.28, p=0.092)	
Yes	0.83 (0.50 to 1.38, p=0.477)	1.10 (0.58 to 1.92, p=0.851)	
Received Family Intervention			
No	Ref	Ref	
Refused	0.94 (0.71 to 1.25, p=0.675)	0.98 (0.61 to 1.65, p=0.988)	
Yes	1.13 (0.71 to 1.81, p=0.612)	0.92 (0.54 to 1.50, p=0.678)	
Received carer-focussed intervention			
Not eligible	Ref	Ref	
No	1.54 (0.84 to 2.83, p=0.168)	1.48 (0.84 to 2.01, p=0.601)	
Yes	1.59 (0.90 to 2.84, p=0.114)	1.24 (0.69 to 1.54, p=0.842)	
Received vocational support			
No	Ref	Ref	
Refused	0.77 (0.48 to 1.25, p=0.293)	0.73 (0.43 to 1.25, p=0.254)	
Yes	1.29 (0.83 to 2.00, p=0.260)	1.29 (0.77 to 2.15, p=0.341)	
Received clozapine			

Not offered	Ref	Ref	Ref
Refused	1.07 (0.68 to 1.68, p=0.762)	1.20 (0.88 to 1.68, p=0.237)	1.09 (0.70 to 1.69, p=0.706)
Yes	**0.67 (0.42 to 0.97, p=0.048)**	0.81 (0.54 to 1.28, p=0.398)	**0.71 (0.45 to 0.94, p=0.039)**
Received intervention for alcohol cessation			
Not required	Ref	Ref	
No	0.94 (0.46 to 1.94, p=0.875)	1.51 (0.47 to 4.91, p=0.491)	
Refused	0.98 (0.41 to 2.33, p=0.959)	0.58 (0.13 to 2.48, p=0.458)	
Yes	**1.94 (1.00 to 3.73, p=0.049)**	1.53 (0.77 to 3.04, p=0.228)	
Received intervention for smoking cessation			
Not required	Ref	Ref	
No	0.55 (0.27 to 1.11, p =0.095)	0.29 (0.08 to 1.10, p=0.069)	
Refused	1.02 (0.62 to 1.68, p =0.928)	0.70 (0.33 to 1.44, p=0.321)	
Yes	**1.73 (1.13 to 2.65, p=0.012)**	1.39 (0.81 to 2.38, p=0.231)	
Received intervention for substance use			
Not required	Ref	Ref	Ref
No	0.86 (0.37 to 1.97, p=0.714)	2.23 (0.48 to 10.42, p=0.308)	0.82 (0.35 to 1.94, p=0.651)
Refused	**2.04 (1.17 to 3.55, p=0.012)**	**2.77 (1.18 to 6.48, p=0.019)**	**1.93 (1.09 to 3.43, p=0.024)**
Yes	**1.92 (1.26 to 2.92, p=0.002)**	1.31 (0.72 to 2.39, p=0.372)	**1.61 (1.04 to 2.51, p=0.033)**
Received intervention for weight loss			
Not required	Ref	Ref	
No	0.78 (0.31 to 1.99, p=0.609)	0.59 (0.18 to 1.96, p=0.392)	
Refused	1.64 (0.65 to 4.12, p=0.297)	2.19 (0.84 to 5.67, p=0.108)	
Yes	1.08 (0.73 to 1.59, p=0.708)	1.18 (0.74 to 1.86, p=0.489)	

* For the continuous variables ‘care coordinator caseload’ and ‘proportion meeting waiting time standard’, stated hazard ratios indicate the change in hazard with a one-unit increase in the exposure. For example, in the unadjusted model each additional person on a care coordinator’s caseload increased the hazard of relapse by 1% (HR 1.01, 95% CI 1.00 to 1.01, p=0.046)

REFERENCES (Appendix)

1. von Elm E, Altman DG, Egger M, Pocock SJ, Gotzsche PC, Vandenbroucke JP, et al. The Strengthening the Reporting of Observational Studies in Epidemiology (STROBE) statement: guidelines for reporting observational studies. *Lancet* 2007; **370**(9596): 1453-7.
2. R Core Team. R: A Language and Environment for Statistical Computing. *R Foundation for Statistical Computing, Vienna, Austria*.
3. Tennant PWG, Murray EJ, Arnold KF, Berrie L, Fox MP, Gadd SC, et al. Use of directed acyclic graphs (DAGs) to identify confounders in applied health research: review and recommendations. *Int J Epidemiol* 2021; **50**(2): 620-32.
4. Burnham K, Anderson D. Model-selection uncertainty with examples. Model selection and multimodel inference: a practical information-theoretic approach. New York, NY: Springer New York; 1998.
5. Austin PC, Steyerberg EW. Events per variable (EPV) and the relative performance of different strategies for estimating the out-of-sample validity of logistic regression models. *Stat Methods Med Res* 2017; **26**(2): 796-808.
